# FOXO1 transcription factor plays a key role in T cell—HIV-1 interaction

**DOI:** 10.1371/journal.ppat.1007669

**Published:** 2019-05-01

**Authors:** Arthur Roux, Héloise Leroy, Bénédicte De Muylder, Lucie Bracq, Samia Oussous, Isabelle Dusanter-Fourt, Ghina Chougui, Rachida Tacine, Clotilde Randriamampita, Delphine Desjardins, Roger Le Grand, Frederic Bouillaud, Serge Benichou, Florence Margottin-Goguet, Remi Cheynier, Georges Bismuth, Marianne Mangeney

**Affiliations:** 1 NSERM, U1016, Institut Cochin, Paris, France; 2 CNRS, UMR8104, Paris, France; 3 Université Paris Descartes, Paris, France; 4 Institut Pasteur Shangai-Chinese Academy of Sciences, Shangai, China; 5 International Associated Laboratory (LIA VirHost), CNRS, Université Paris Descartes, Institut Pasteur Paris, and Institut Pasteur Shangai-Chinese Academy of Sciences, Shangai, China; 6 CEA, Université Paris Sud, INSERM -Immunology of Viral Infections and Autoimmune Diseases department (IMVA), U1184, IDMIT Department, Fontenay-aux-Roses, France; Imperial College London, UNITED KINGDOM

## Abstract

HIV-1 is dependent on the host cell for providing the metabolic resources for completion of its viral replication cycle. Thus, HIV-1 replicates efficiently only in activated CD4^+^ T cells. Barriers preventing HIV-1 replication in resting CD4^+^ T cells include a block that limits reverse transcription and also the lack of activity of several inducible transcription factors, such as NF-κB and NFAT. Because FOXO1 is a master regulator of T cell functions, we studied the effect of its inhibition on T cell/HIV-1 interactions. By using AS1842856, a FOXO1 pharmacologic inhibitor, we observe that FOXO1 inhibition induces a metabolic activation of T cells with a G0/G1 transition in the absence of any stimulatory signal. One parallel outcome of this change is the inhibition of the activity of the HIV restriction factor SAMHD1 and the activation of the NFAT pathway. FOXO1 inhibition by AS1842856 makes resting T cells permissive to HIV-1 infection. In addition, we found that FOXO1 inhibition by either AS1842856 treatment or upon FOXO1 knockdown induces the reactivation of HIV-1 latent proviruses in T cells. We conclude that FOXO1 has a central role in the HIV-1/T cell interaction and that inhibiting FOXO1 with drugs such as AS1842856 may be a new therapeutic shock-and-kill strategy to eliminate the HIV-1 reservoir in human T cells.

## Author summary

HIV-1 is controlled by host restriction factors that interfere with its life cycle. However, the virus has equipped itself to counter these strategies. We report a new interplay between HIV-1 and human T lymphocytes through the FOXO1 transcription factor. By using AS1842856, a drug targeting FOXO1, we found that FOXO1 inhibition triggers metabolic activation and G_0_/G_1_ transition of resting T cells and also by the inactivation of the SAMHD1 viral restriction factor. FOXO1 inhibition makes resting CD4^+^ T cells permissive to HIV-1 infection. We finally found that pharmacologic (AS1842856 treatment) or genetic (shRNA) silencing of FOXO1 reactivate HIV-1 latent proviruses. Thus FOXO1 appears as an important player of the HIV-1/T-cell relationship and a new potential therapeutic target for intervention during HIV-1 infection.

## Introduction

As other viruses, HIV-1 is an obligate intracellular pathogen strictly dependent on a suitable host cell machinery for most of the steps of its life cycle, a machinery that is hijacked by the virus to generate its progeny. In the case of human CD4^+^ T lymphocytes, the permissiveness to HIV-1 infection depends on their cellular activation state. While activated and proliferating CD4^+^ T lymphocytes are highly susceptible to infection and support efficient HIV-1 replication, resting CD4^+^ T cells are mainly non-permissive because of their low level of transcriptional activity [1]. Host cell transcription factors such as NF-κB and NFAT, the activity of which is dependent on T cell stimulation, recognize specific target sites in the viral promoter contained in the long terminal repeats (LTRs), and are therefore essential for expression of viral components and HIV-1 genome replication [2]. This transcriptional control is also instrumental for the generation of viral reservoirs, defined as cell types where the virus persists during therapy [3,4]. These reservoirs, established during the first days of infection, are responsible for the recurrence of a detectable level of viremia in treated patients upon interruption of combinatory antiretroviral therapy (cART). The main reservoir resides in latently infected resting CD4^+^ memory T cells [5]. These cells carry stably integrated and transcriptionally silent but replication-competent proviruses. They do not produce virus particles when cells are in a resting state, but can give rise to infectious virions following activation by various stimuli, leading to viral rebound when cART is interrupted.

In T cells, IL-7 is critical for the loss of quiescence [6,7]. In this context, primary naive T cells, typically not permissive to HIV, can be productively infected when pre-treated with IL-7 alone [8,9]. One molecular step that participates in this effect of IL-7 is the neutralization of SAMHD1 activity [10]. SAMHD1 is one of the cellular factors that have evolved to counteract HIV-1 replication. These so-called restriction factors constitute barriers present in the host cell to inhibit specific steps of the viral life cycle. SAMHD1 is a deoxynucleoside triphosphate triphosphohydrolase that regulates cell-cycle progression, and is a major viral restriction factor that blocks early reverse transcription of HIV-1 by depleting the intracellular dinucleotide triphosphate (dNTP) pool [11,12]. The function of SAMHD1 is regulated through the phosphorylation of threonine 592 by cyclin A2/Cdk1, an event that is induced by IL-7 [13]. Previous work also showed that IL-7 induced NFAT activity is a supplementary mechanism through which IL-7 can affect HIV-1 infection in naïve T cells [8].

Thus, it is now well established that the mechanisms that control the state of quiescence of naïve T cells are essential for regulating their permissiveness to HIV infection [1]. FOXO1 is a transcription factor that actively maintains quiescence of human T lymphocytes in conditions where the PI3-kinase/Akt pathway is inactive [14]. Data showing an increase of viral replication kinetics after inhibition of FOXO1 in quiescent T cells treated with IL-7 now suggest that this molecule may be another molecular switch controlling HIV-1 infection and participating in the effects of this cytokine on the biology of HIV-1 in T cells [15]. In this study, we explored whether and how directly inhibiting FOXO1 activity with AS1842856 [16], a specific pharmacological inhibitor of FOXO1 affects the permissiveness of naïve human T cells to HIV infection. We show that inhibition of FOXO1 alone was sufficient to trigger a G_0_→G_1_ transition of human T lymphocytes upstream of the R restriction point of the cell cycle. This transition is characterized by a parallel increase in cell size, metabolism and transcriptional activity. We also show that FOXO1 inhibition is accompanied by the inactivation of the SAMHD1 viral restriction factor together with permissiveness of resting human CD4^+^ T cells to lentiviral infection. We finally observe the reactivation of HIV-1 proviruses by the AS1842856 drug or after FOXO1 knowdown by RNA interference using different HIV-1 latency models of human T cells, and also of latent viral reservoirs present in CD4^+^ T cells from nonhuman primates under cART. Taken together, these results demonstrate that FOXO1 is a major player in T lymphocyte/HIV-1 interaction and that its pharmacological inhibition is a new potential clinical strategy to eradicate latent provirus reservoirs during HIV-1 infection.

## Results

### FOXO1 inhibition alone by AS1842856 allows HIV-1 infection of resting T cells

**Fig 1:**
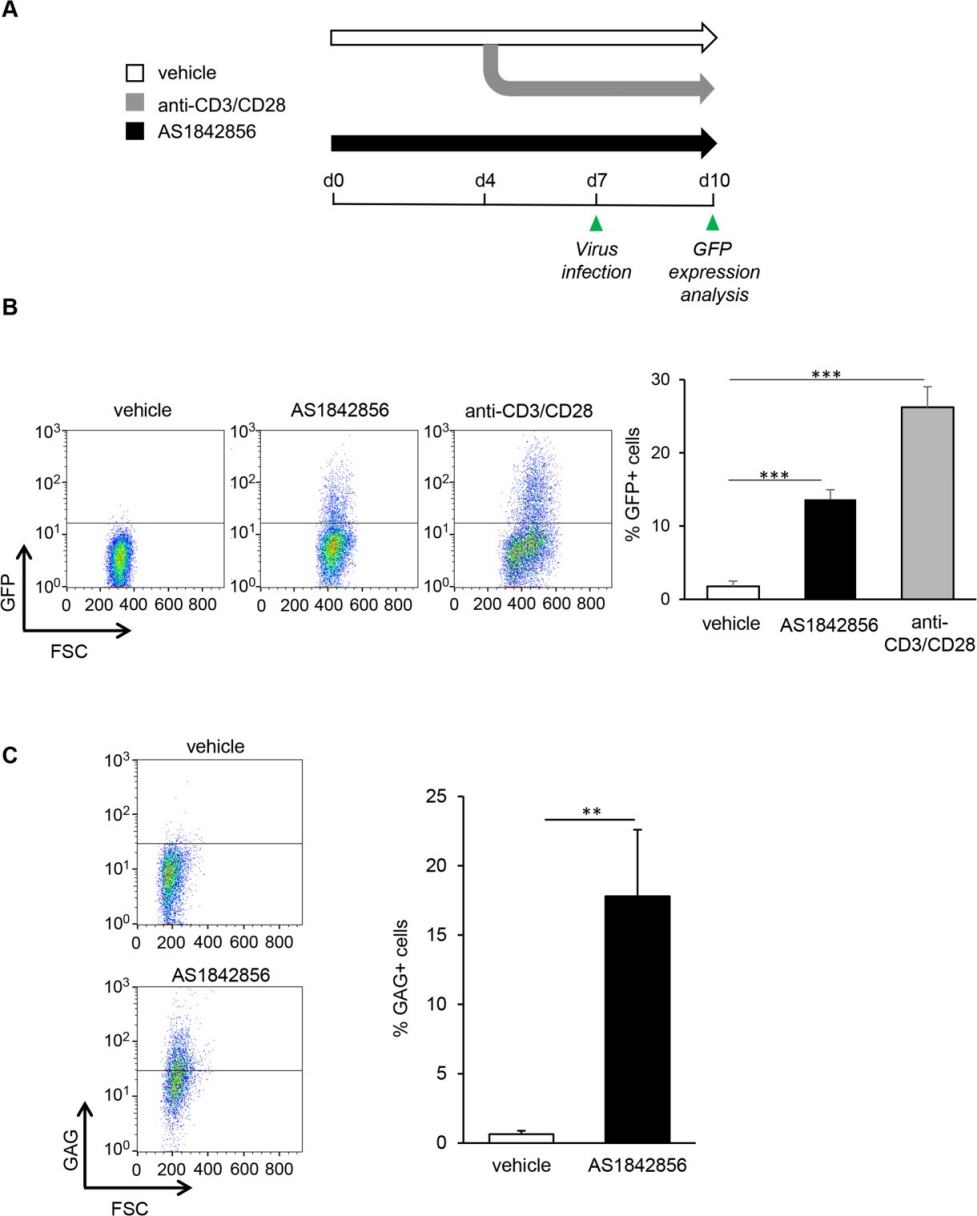
AS1842856 allows HIV-1 infection of human resting T cells. (A) Experimental design. After 7 days of culture with AS1842856 (500nM) or vehicle only, PBT were infected with a pseudotyped HIV-1 retrovirus encoding GFP. 3 days later GFP fluorescence in the viable cells was analyzed by FACS. Untreated T cells activated during 3 days with anti-CD3/CD28 beads before infection were used as a positive control. (B) Results obtained with one representative donor are shown in the left panel and mean results, +/- SE, with cells from five different donors in the right panel. (C) PBT treated 7 days with AS1842856 (500nM) or vehicle only were infected with the HIV-1 strain NL4.3. After 3 days of infection, GAG expression was measured by FACS using a GAG-specific antibody (left panel). Mean results +/- SE with cells from 3 different donors are shown in the right panel.

We first determined whether FOXO1 inhibition by AS1842856 allowed the infection of resting T cells by HIV-1, in the absence of any additional treatment. For this aim, peripheral blood human T cells (PBT) were cultured with AS1842856 or DMSO vehicle control only, and then brought into contact with a VSV-G non-replicative lentiviral vector expressing GFP under LTR control. Three days later, the percentage of GFP-positive cells was analyzed by flow cytometry. As a positive control, we also examined the infection of PBT stimulated with anti-CD3/CD28 beads. A view of the experimental schedule is given in Fig 1A. FACS dot plot analyses of the results obtained with a representative donor, as well as mean results from five independent donors, are illustrated in Fig 1B (left and right panel, respectively). They show a marked increase of GFP positive cells after FOXO1 inhibition. Since the use of V-SVG envelope to infect resting T cells can introduce a bias in these experiments, we next checked using the same experimental set-up the capacity of AS1842856-treated PBT to be infected with a *bona fide* HIV-1 strain, NL4.3. Three days after infection by NL4.3, intracellular expression of the GAG precursor was measured by flow cytometry. As shown in Fig 1C, the number of GAG positive cells increases after AS1842856 treatment, and dose-dependently (S1 Fig, upper panel). Similar results were obtained with the LAI HIV-1 strain, thereby demonstrating that the transactivation induced by AS1842856 was not restricted to NL4.3 viruses (S1 Fig, lower panel). Thus inhibition of FOXO1, in the absence of any other cell treatment, allows infection of resting T cells by HIV-1.

### Inhibition of FOXO1 activity increases T cell metabolism

**Fig 2:**
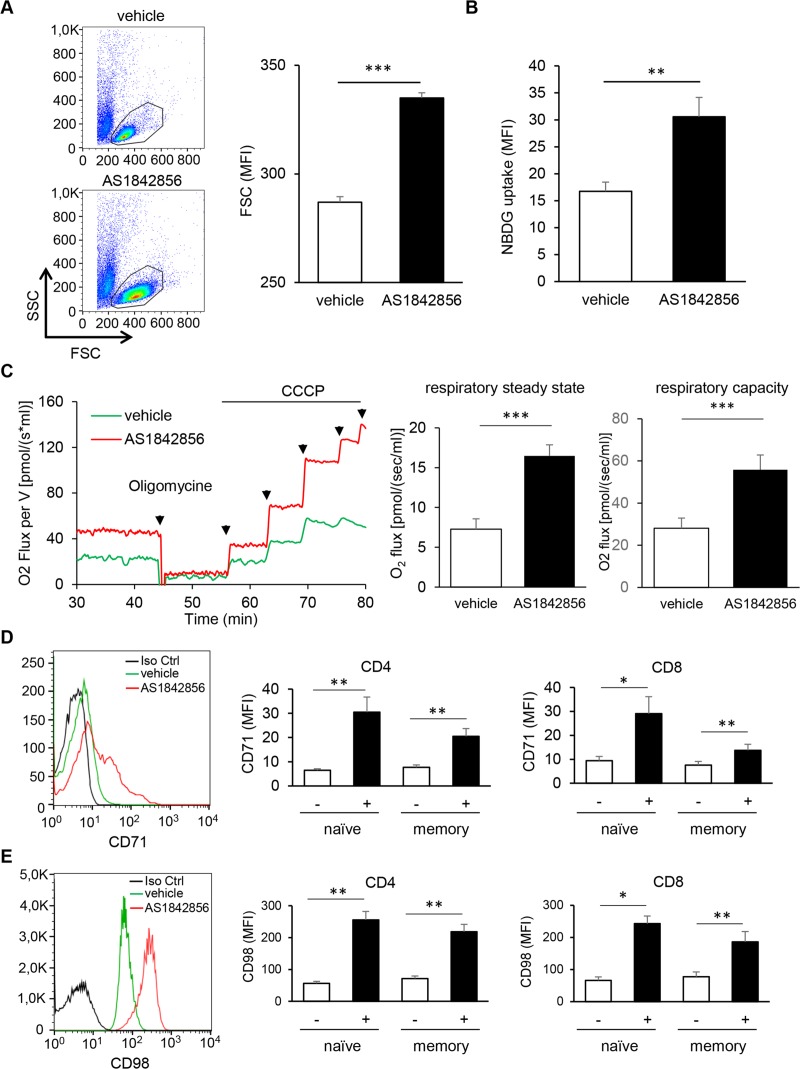
AS1842856 induces a substantial increase in T-cell metabolism. (A) PBT were cultured for 7 days with 500nM of AS1842856 or vehicle only and their cell size (FSC) analyzed by FACS. Mean results +/- SE with T cells from six independent donors are shown in the right panel. (B) Glucose uptake of T-cells treated with AS1842856 (500nM) for 7 days or vehicle only was measured by FACS after labelling with 2-NBDG. Mean results +/- SE with T cells from four independent donors are shown. (C) High-resolution respirometry of living T-cells (25.10^6^/ml) treated with AS1842856 (500nM) for 7 days or vehicle only was measured using an Oxygraph-2k instrument (Oroboros Instrument). Routine respiration (respiratory steady state) was first measured, followed by the addition of Oligomycin (1μM) to inhibit ATP synthase, reducing respiration to a baseline leak state. Successive CCCP (carbonyl cyanide m-chlorophenyl hydrazine) titrations were then used to stimulate respiration to the non-coupled state of electron transfer capacity (giving the maximum respiratory capacity). Mean results +/- SE (normalized to 10.10^6^ cells/ml) of routine respiratory and maximum respiratory capacity with T cells from 5 independent donors are shown in the right panels. (D and E) CD71 and CD98 expression were measured by FACS with biotinylated specific antibodies followed by streptavidin-PE labeling in PBT exposed or not (vehicle only) to AS1842856 (500nM) for 7 days. Histograms in the left panels show expression in the whole T-cell population for one representative donor. A biotinylated anti-CD19 antibody was used as a negative control (black histograms). Shown in the right panels are the expression of CD71 and CD98 after the same treatment measured in CD4^+^ and CD8^+^, naïve and memory T-cell subpopulations, distinguished after labeling with CD4, CD8 and CD45RA-specific antibodies. Mean results +/- SE obtained with 7 and 4 different donors for CD71 and CD98, respectively, are shown.

Retrovirus replication is highly dependent on the metabolic activity of the cellular host [17,18]. We therefore hypothesized that the susceptibility to HIV-1 infection of FOXO1-inhibited resting T cells could be due to an increased cell metabolism. Cell size variation is often linked to metabolic rate. As shown in Fig 2A, AS1842856 induces a substantial increase of T cell size, illustrated by FSC/SSC dot plot analyses. Time-course analyses showed a gradual and continuing increase, usually reaching a maximum after 7 days of culture (S2A Fig) and for drug concentrations around 500nM (S2B Fig). Importantly, no associated toxicity of the drug was observed (S2C Fig*)*. These results led us to use this condition (500nM during a 7-day culture) in all subsequent experiments. Parallel labelling of CD4^+^ and CD8^+^ T-cells and of their naïve (CD45RA+) and memory (CD45RA-) sub-populations showed that this cell size increase was very similar in both CD4^+^ and CD8^+^ T-cell subsets (S2D Fig). They also indicated that within these two subsets both naïve and memory T cells were similarly affected.

As increased cell metabolism is often associated with glucose consumption, we analyzed the uptake of the fluorescent glucose analog 2-NBGD in T cells treated or not with AS1842856. As shown in Fig 2B, FOXO1 inhibition induced a significant increase of 2-NBDG uptake. We also checked the consequences of AS1842856 treatment on mitochondrial respiration, another cell function associated with an increase in metabolism. Results obtained by high-resolution respirometry experiments of PBT treated with or without AS1842856 showed that respiration at the steady state was increased by AS1842856 (Fig 2C). Using oligomycin, an inhibitor of ATP synthase which reduces respiration to the baseline leak level, followed by successive addition of CCCP (carbonyl cyanide m-chlorophenyl hydrazone) to stimulate respiration to the non-coupled state of the electron transfer capacity, we also observed that the maximum respiratory capacity was strongly increased by the drug. Finally, we investigated the effect of AS1842856 on the expression of the receptor of transferrin (CD71) (Fig 2D) and the heavy chain of the system L amino-acid transporter (CD98) (Fig 2E). These cell-surface markers are known to be associated with an increased metabolic status in T lymphocytes [19–22]. Mirroring the glucose uptake and mitochondrial respiration results, we observed a significant increase of these two receptors on T cells treated with AS1842856. Both CD4^+^ and CD8^+^ T-cells and their naïve and memory subsets were affected.

**Fig 3:**
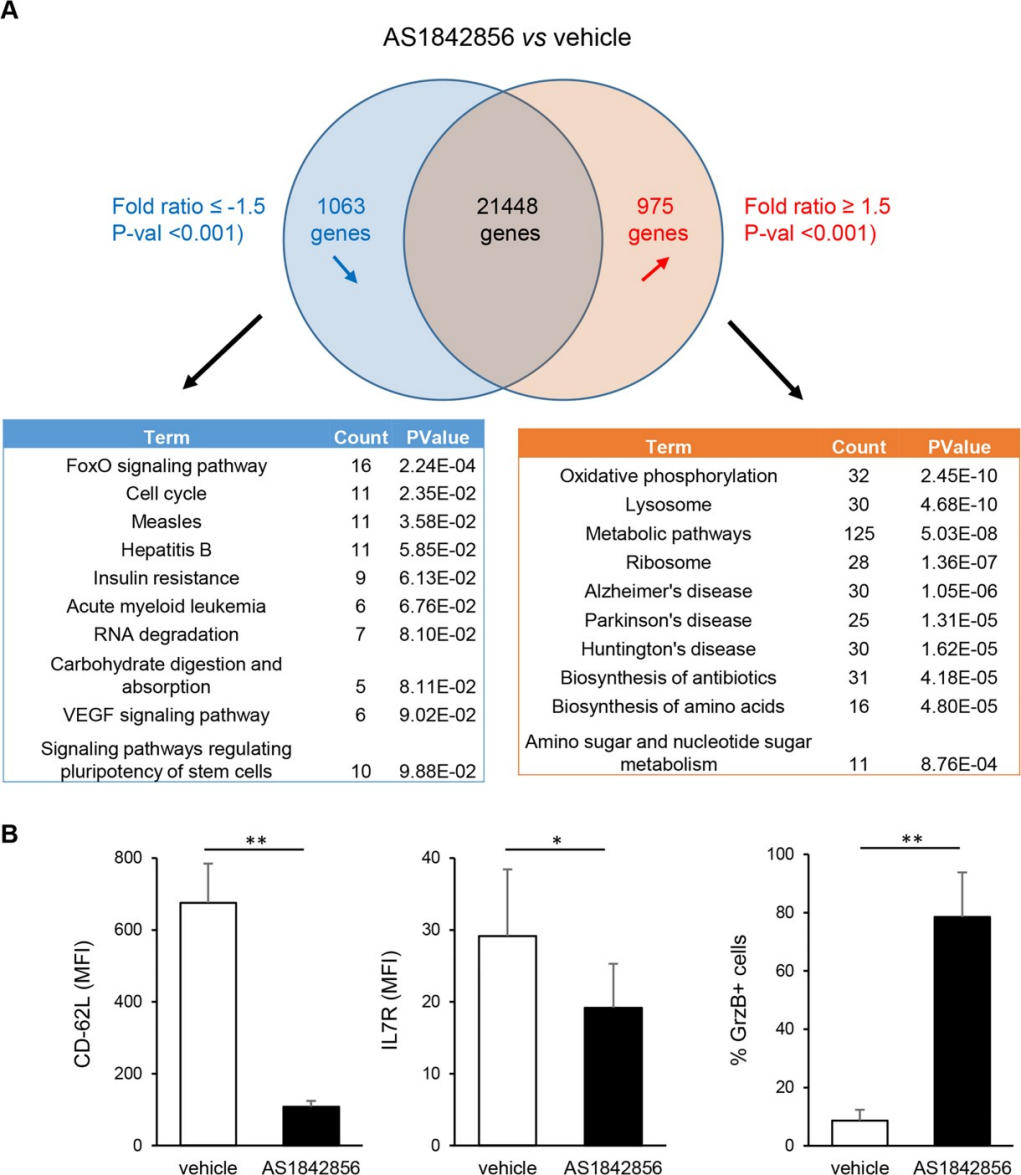
Transcriptome analysis of PBT exposed to AS1842856 treatment. (A) Microarray analyses of PBT from 3 individual donors cultured during 7 days in the presence of the drug (500 nM) or vehicle only were performed. Microarray CEL files were analyzed using the Transcriptome Analysis Console software (ThermoFisher Scientific). On the basis of a <-1.5 and >1.5-fold change cut-off and a P-val <0.01, 1063 genes were down-regulated by AS1842856 and 975 genes were up-regulated. Analysis of these two gene lists using the functional annotation tool of the DAVID Bioinformatics Resources and the KEGG database is also shown, with the most significant pathways altered after AS1842856 treatment. (B) CD62-L, IL-7R and Granzyme B expression were measured by FACS with specific antibodies in PBT exposed to AS1842856 (500nM) or vehicle only during 7 days. Mean results +/- SE obtained with 4 different donors are shown.

To get an overall view of these changes in T-cell metabolism, gene expression microarray analysis of PBT cultured during 7 days in the presence or not of AS1842856 were performed. By comparing the results obtained from 3 individual donors, lists of mRNAs whose levels were down-regulated or up-regulated after AS1842856 treatment (with a <-1.5 and >1.5-fold change cut-off and a P-val <0.01) were established. Each contains around 1000 differentially expressed genes (S1 Table). These gene lists were analyzed using the functional annotation tool of the DAVID Bioinformatics Resources [23,24] and the KEGG database. Results identified FoxO signaling genes and genes involved in the negative regulation of the cell cycle as the most significantly inhibited by AS1842856 (Fig 3A). In contrast, AS1842856-treated cells showed a strong increase in the expression of molecular networks involved in cell metabolic activity. Among them, and in accordance with the mitochondrial respiration results, the oxidative phosphorylation pathway was the most affected. We also verified at the protein level that some prototypic targets of FOXO1 in T cells whose expression is positively or negatively controlled by FOXO1, such as CD62-L and IL7-R [25] or granzyme B [26,27], respectively, were also down or up-regulated after AS1842856 treatment (Fig 3B).

### Inhibition of FOXO1 induces a transition from quiescence (G_0_) to the G_1_ phase of the cell cycle

**Fig 4:**
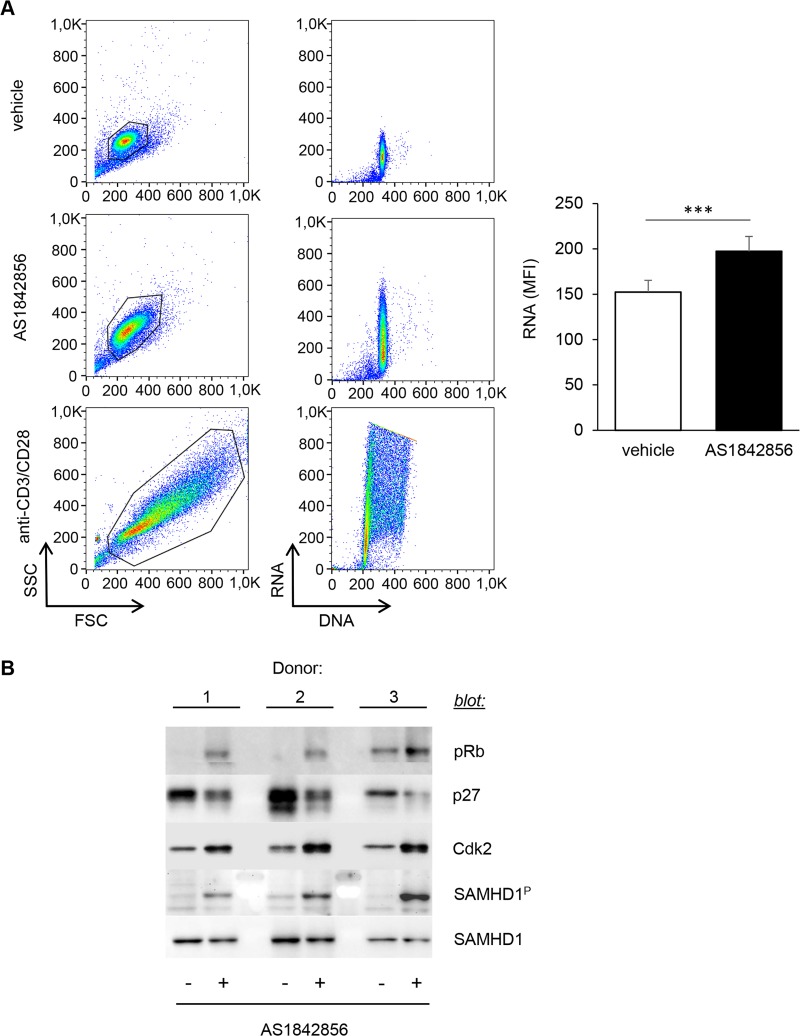
AS1842856 leads to a transition from quiescence to the G_1_ phase of the cell cycle of T lymphocytes. (A) PBT were cultured for 7 days in the presence of AS1842856 (500nM) or vehicle only. RNA and DNA cell content was measured by FACS on the gated cells after acridine orange staining. Untreated T cells activated for 3 days with anti-CD3/CD28 beads were used as a positive control of cells increasing both their RNA and DNA content. Result obtained with one representative donor (left panel) and mean results +/- SE with cells from 7 independent donors (right panel) are shown. (B) PBT from 3 independent donors were cultured as in A, then collected, lysed and immunoblotted using specific antibodies against p27, Cdk2 and SamHD1 proteins. The same lysates were also analyzed for Rb and SAMHD1 phosphorylation with phospho-specific antibodies.

Increase in cell size and number of organelles (such as mitochondria), as well as accumulation of nutrients, are hallmarks of the transition from the G_0_ to the G_1_ phase of the cell cycle that are required to prepare the subsequent phases leading to mitosis [28]. Moreover, the transcriptome modification induced by AS1842856 treatment of PBT revealed that the cell cycle pathway was one of the most affected (Fig 3A and S1 Table). We therefore directly investigated the cell cycle status of PBT treated with AS1842856 using acridine orange staining, an intercalating dye that labels both RNA and DNA. As a positive control of increase in both RNA and DNA cellular content, we used untreated T cells activated for 3 days with anti-CD3/CD28 beads. Results showed that AS1842856 markedly increased cellular RNA levels without any significant change of the DNA content, whereas CD3/CD28 beads increased both (Fig 4A). This was confirmed by classical CFSE dilution assays (S3 Fig). These results demonstrate that AS1842856-treated PBT show characteristic features of cells undergoing a G_0_→G_1_ cell cycle progression, but without any cell division.

To further investigate this process, we checked whether typical molecular events involved in cell progression through the G_1_ phase of the cell cycle, such as Rb phosphorylation, p27 down-regulation or CDK2 up-regulation [29], were changed after AS1842856 treatment. In parallel to an increase of Rb phosphorylation, we observed a decrease in p27 expression, paralleled by an up-regulation of CDK2 (Fig 4B) (also found at the mRNA level, see S1 Table). As phosphorylation of the retroviral restriction factor SAMHD1 (*i*.*e*. its inactivation) by CDK2 is associated with the exit of the quiescent state and also because this molecular event controls T-cell susceptibility to HIV-1 infection [13], we also measured pSAMHD1 levels after AS1842856 treatment of PBT. A clear phosphorylation was consistently found (Fig 4B). This phosphorylation was less pronounced than after CD3/CD28 stimulation, which is known to strongly trigger SAMHD1 phosphorylation in T cells [10] (S4 Fig). Additionally, in parallel experiments measuring the permissiveness of AS1842856 treated cells to HIV-1 infection, we also observed a relationship between SAMHD1 phosphorylation levels and GAG expression (S5 Fig).

### AS1842856 potentiates LTR activity

**Fig 5:**
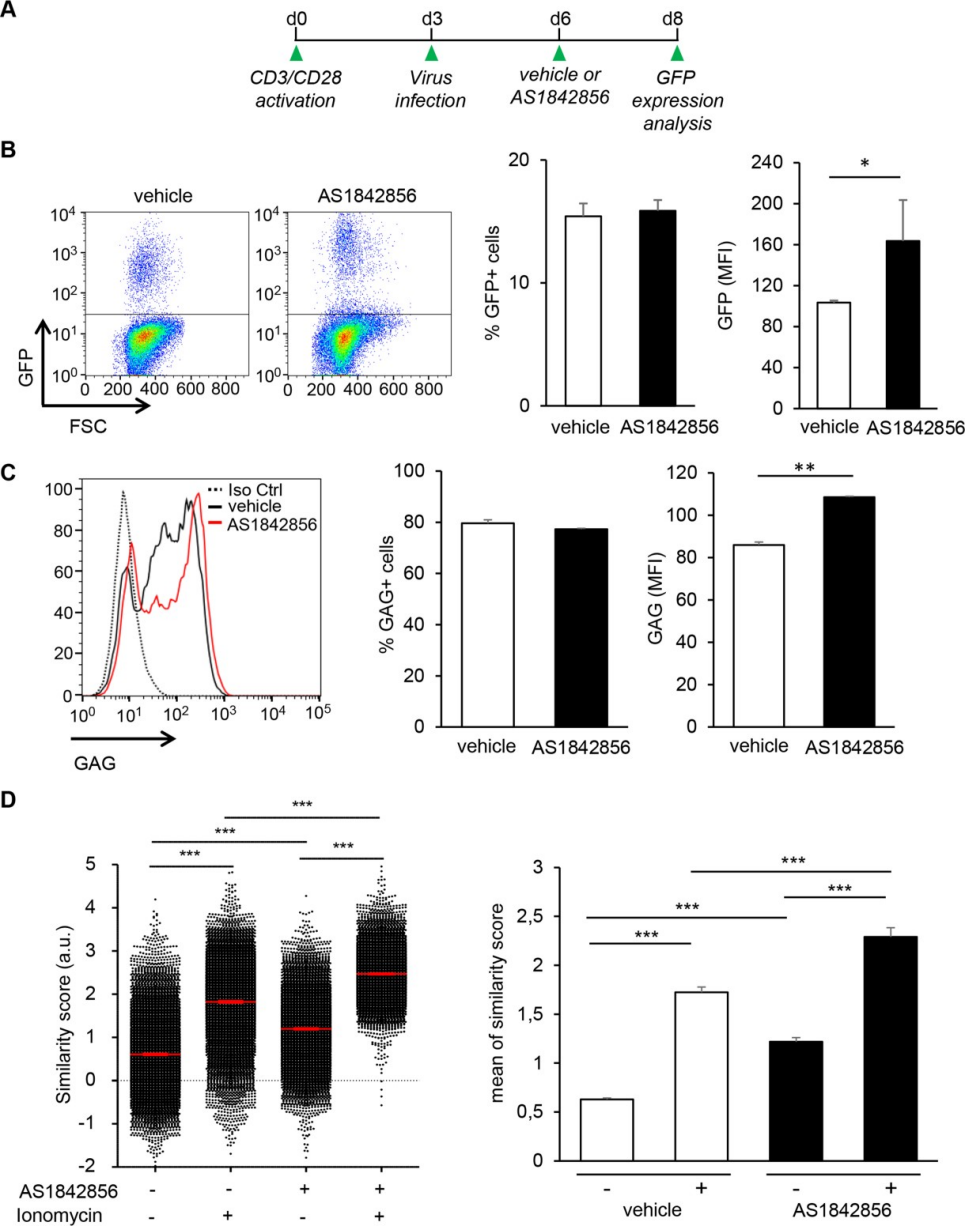
AS1842856 is a potent activator of HIV-1 LTR in human T cell. (A) Experimental design. PBT were stimulated for 3 days with anti-CD3/CD28 beads and then infected with a pseudotyped HIV-1 retrovirus encoding GFP. Three days after infection, AS1842856 (500nM) or vehicle only were added to the culture. GFP expression levels were measured by flow cytometry two days later. (B) Result obtained with one representative donor (left panel) and mean results +/- SE of GFP expression measured in the GFP-positive gated cell population of 4 different donors (right panel) are shown. (C) Chronically HIV-1 infected H9 cells were cultured three days with AS1842856 or vehicle only. At the end of culture, GAG expression level was analyzed by flow cytometry. Result obtained in one experiment (left panel) and mean results +/- SE of 3 independent experiments (right panel) are shown. (D) ImageStream analysis of nuclear localization of NFAT1 in PBT treated for 7 days with AS1842856 (500nM) or vehicle only, either at the steady state, or after a 30 min stimulation with the calcium ionophore ionomycin. Results obtained with one representative donor are shown in the left panel, with the red bars representing the mean +/- SE of similarity score of at least 3x10^3^ individual cells (black dots) in each experimental condition. Mean +/- SE of these similarity scores obtained with 3 different donors are shown in the right panel.

Since HIV-1 replication in resting T cells is limited by the transcriptional activity of the viral LTR, we also investigated the consequences of FOXO1 inhibition on LTR activity (*i*.*e*. at the post-integrative level). For this purpose, PBT were stimulated with anti-CD3/CD28 beads and then infected with the previously used VSV-G non-replicative lentiviral vector expressing GFP. Subsequently, the cells were incubated with or without AS1842856 for two days, and GFP expression levels measured by flow cytometry to see whether FOXO1 inhibition by the drug could activate the LTR integrated in the host cell genome. A representation of the experimental schedule is given in Fig 5A. Results showed that whereas the percentage of GFP-positive cells remained unchanged, there was a marked increase in GFP fluorescence intensities in the presence of AS1842856 in these cells, as compared to the control (Fig 5B). We concluded that AS1842856 could increase LTR activity in the absence of any other viral proteins. In order to validate this result in a model where all viral proteins are present, we also used chronically HIV-1 infected T-lymphoid H9 cells, a clonal derivative of the Hut 78 lymphoma T-cell line. These cells were treated with AS1842856 for 3 days and GAG expression analyzed by flow cytometry. As shown in Fig 5C, as expected, a high fraction of these cells spontaneously expressed GAG. However, in this model also, AS1842856 treatment increased LTR activity, as illustrated by the clear shift in GAG expression.

LTR activity is mainly controlled by NFAT and NF-κB, which transcriptional activities are dependent on T cell activation. We therefore measured the activity of these transcription factors in PBT after AS1842856 treatment. No activation of the NF-κB pathway by AS1842856 could be detected, given the absence of degradation of the NF-κB inhibitor IκBα (S6 Fig). A short PMA plus iomycin stimulation was used here as a positive control, showing an almost complete loss of IκBα, also seen with cells that have been pretreated with AS1842856. In contrast, we observed a clear nuclear translocation of NFAT1 in AS1842856-treated cells (Fig 5D). In this experiment, we also observed that the drug potentiated the effect of the calcium ionophore ionomycin, initially used as a positive control to trigger NFAT1 activation by increasing intracellular calcium. In a consistent way, we found in parallel experiments that steady-state levels of intracellular calcium were higher in AS1842856-treated cells (S7A Fig) and that the drug could also potentiate the response to ionomycin (S7B Fig).

### FOXO1 inhibition reactivates latent forms of HIV-1

**Fig 6:**
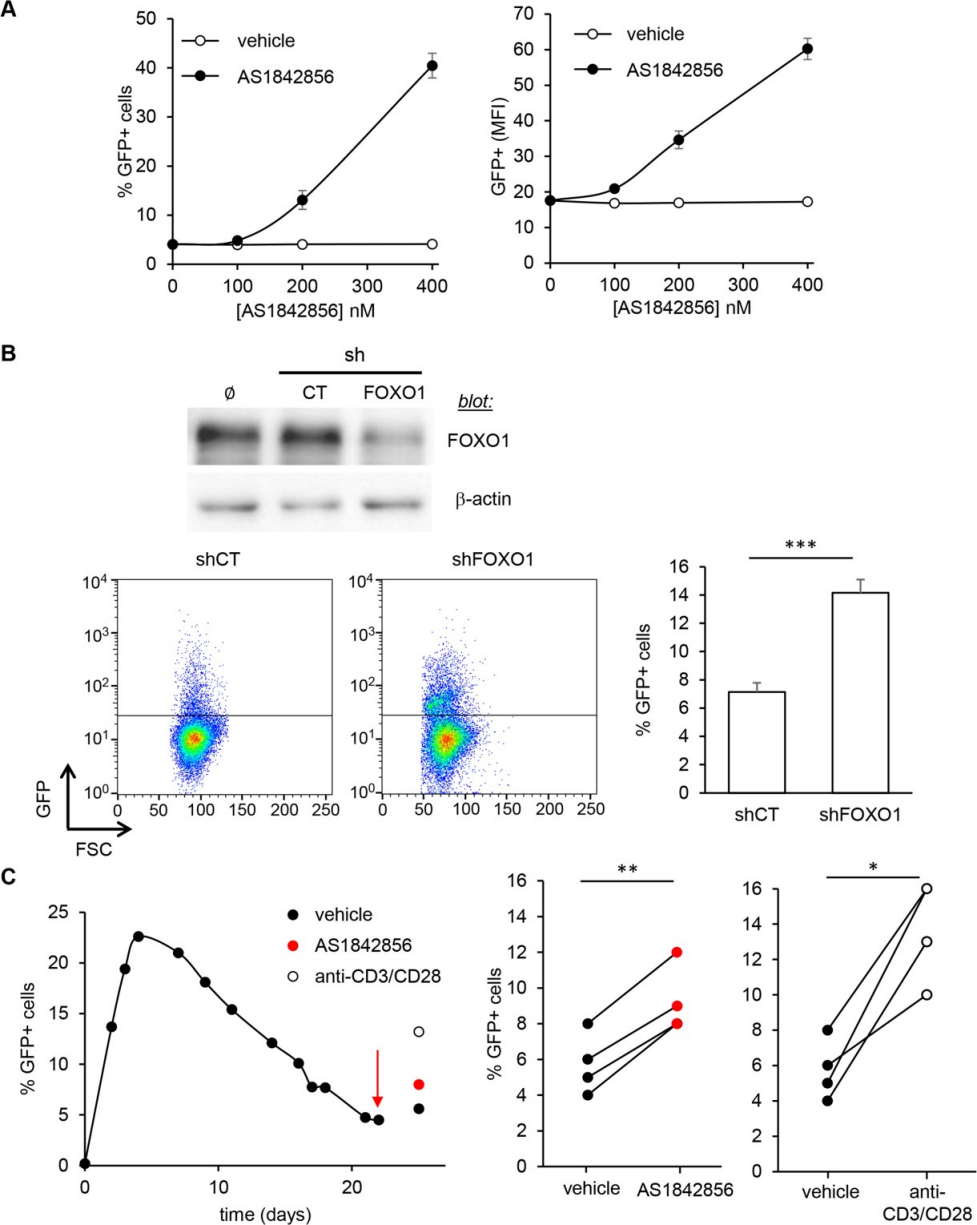
FOXO1 inhibition allows HIV-1 latent provirus reactivation *in vitro*. (A) J-Lat A1 cells were treated with 400nM of AS1842856 or vehicle only at different concentrations and GFP expression measured by FACS after 2 days of culture. % of GFP-positive cells (left panel) and mean GFP intensity of these GFP-positive cells (right panel) are shown. Mean results +/- SE from 5 independent experiments are shown. (B) FOXO1 expression in J-Lat A1 cells after transduction with control luciferase (CT) or FOXO1 shRNA encoding retroviral particles was measured by western blot (upper panel). GFP expression was measured by FACS in these cells 5 days post-transduction. A representative experiment is shown in the left panel and the results obtained from 3 independent experiments are shown in the right panel. (C) PBT were activated with anti-CD3/anti-CD28-coated beads and infected 3 days later with a VSV-G LTR-GFP-LTR retrovirus and maintained in the presence of IL2 (20 UI/ml). The percentage of GFP-positive cells was then followed by FACS at different time points. Cells were then treated (arrow) with the vehicle only, AS1842856 (500nM) or anti-CD3/anti-CD28-coated beads as a positive control. Latency reversal was assessed by measuring GFP fluorescence after 3 days of reactivation. A representative experiment is shown in the left panel. Shown in the right panels are the results obtained from 4 independent experiments with different donors after the 3-day reactivation period.

To further study the consequences of FOXO1 inhibition on LTR activity, and especially to explore the ability of AS1842856 to reactivate latent forms of HIV-1, we next used the J-Lat cell line HIV-1 latency model system. J-Lat cells were derived from the leukemia T cell line Jurkat. They contain an integrated silent form of a minimal HIV-1 provirus encoding GFP that can be used as a fluorescent read-out of the reactivation of the latent provirus [30]. In various cell types, one main mechanism involved in FOXO1 inhibition by AS1842856 results from the direct inhibition by the drug of FOXO1 transcriptional activity [16]. Thus, we first checked whether the same mechanism held true in Jurkat cells. For this aim we used a dual-luciferase reporter assay system with a reporter plasmid controlled by the Forkhead responsive element [31]. Cells were co-transfected with vectors encoding either GFP or the constitutively active form of FOXO1, mutated on the three phosphorylation sites by Akt, FOXO1TM GFP. As shown in S8A Fig, AS1842856 treatment strongly inhibits the transcriptional activity of FOXO1TM. An inhibition was also observed in cells transfected with the GFP control vector, suggesting an inhibition by the drug of the residual activity of the endogenous form of FOXO1 in this cell line. In agreement, we found that the strong expression of CD62-L triggered by FOXO1TM GFP in this T-cell line, was also markedly inhibited by AS1842856 dose-dependently (S8B Fig). Again, this experiment suggested some residual activity of the endogenous form of FOXO1 to control CD62-L levels in Jurkat cells, as its expression was also decreased by AS1842856 in cells transfected with GFP alone. After having checked this, J-Lat cells (clone A1) were incubated with different concentrations of AS1842856. After a 3-day treatment we observed a strong dose-dependent increase of the percentage of GFP-positive cells, as well as an increase of GFP expression levels, indicative of a reactivation of the LTR (Fig 6A). These results were confirmed with two other J-Lat cell clones (S9 Fig). To strengthen these observations, we measured reactivation induced by a non-pharmacological approach by knocking-down FOXO1 expression in the J-Lat A1 clone using a FOXO1-specific shRNA construct. These cells showed an increase percentage of GFP-positive cells, as compared to cells in which a control shRNA had been used (Fig 6B).

We next investigated whether these findings could be extended to primary T cells. For this purpose, we set up an experimental model using PBT activated with anti-CD3/anti-CD28-coated beads, then infected with the previously used (see Fig 1A) VSV-G non-replicative lentiviral vector expressing GFP under LTR control. Cells were maintained in culture with interleukin 2 (IL-2) for several weeks. As shown in Fig 6C (left panel), the percentage of GFP-positive cells continuously decreased over time, due to a gradual silencing of LTR activity, as reported previously [32]. Cells were then treated with AS1842856 or anti-CD3/anti-CD28-coated beads as a positive control, and latency reversion was assessed by measuring GFP fluorescence after 3 days of reactivation. AS1842856 treatment was found to increase the number of GFP-positive cells (Fig 6C, left panel). Repeating these experiments with 4 donors, we observed that, although lower than the reactivation induced by anti-CD3/anti-CD28 beads, a significant increase of virus reactivation was always found with AS1842856 (Fig 6C, right panels). These results demonstrate that inhibiting FOXO1 with AS1842856 could reverse HIV-1 latency in human T lymphocytes.

### AS1842856 reactivates latent SIVmac in T cells from non-human primates under cART treatment

**Fig 7:**
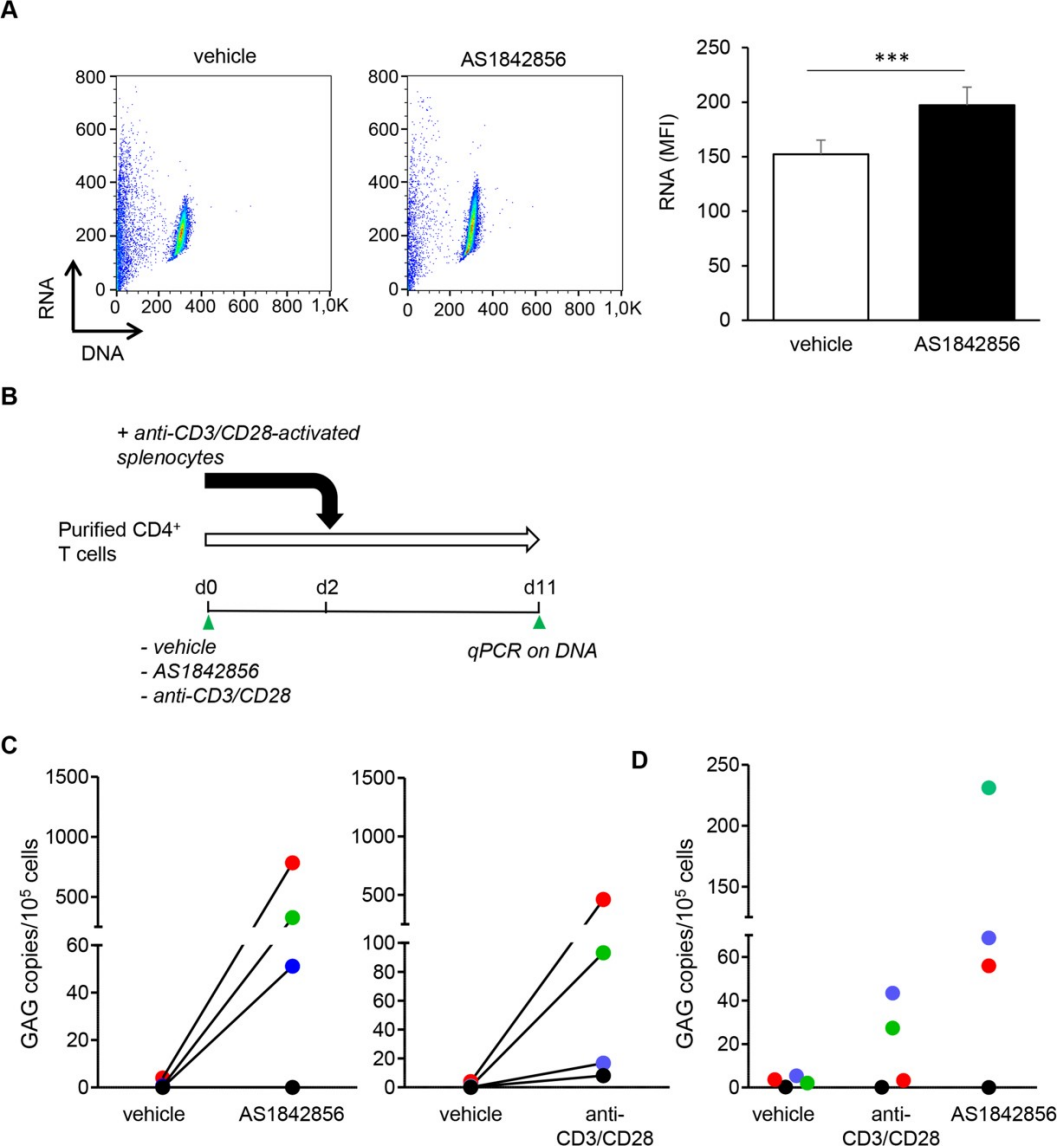
AS1842856 allows latent provirus reactivation *ex vivo*. (A) PBT purified from healthy macaques were cultured for 7 days in the presence of AS1842856 (500nM) or vehicle only. RNA and DNA cell content were measured by FACS on the gated cells after acridine orange staining. Results obtained with one representative macaque (left panel) and mean results +/- SE with T cells from 3 independent animals (right panel) are shown. (B) Experimental design. CD4^+^ T cells from animals infected with SIV mac251, and treated for 6 months with antiretroviral agents (Tenofovir, Emtricitabine, Dolutegravir), were cultured for two days with AS1842856 (500nM), vehicle only or anti-CD3/CD28 coated beads, used here as positive control. Heterologous simian splenocytes from non-infected animals stimulated for three days with anti-CD3/CD28 beads were then added. Measurements by quantitative PCR of *Gag* sequence in genomic DNA were performed nine days later. (C) Results obtained with four animals, each represented by a distinct color symbol, are shown. (D) Supernatants harvested at the end of the above assays were ultracentrifugated and concentrated viruses added to 10^6^ heterologous splenocytes from non-infected monkey preactivated with anti-CD3/CD28 beads. After 5 days of culture, cells were harvested and infection levels were measured by *Gag* sequence quantification in genomic DNA. The same color code than in panel C was used for each animal.

In order to confirm this result in a model more relevant to pathophysiology, we investigated whether AS1842856 could reactivate latent SIVmac in CD4^+^ T cells from non-human primates under cART treatment. For this aim, we used rhesus macaques that had been previously infected by SIV mac251, and treated for 6 months with a triple antiretroviral therapy combining Tenofovir, Emtricitabine and Dolutegravir to induce latency. We first controlled that, as in human T cells, AS1842856 was able to induce the G_0_→G_1_ transition of T cells purified from the blood of healthy macaques (Fig 7A*)*. Next, CD4^+^ T cells from the blood of the infected macaques were purified and cultured with AS1842856, anti-CD3-CD28 coated beads as a positive control, or vehicle only. Two days later, to amplify infectious viruses produced by CD4^+^ T cells, activated splenocytes from non-infected macaques were added. Nine days later genomic DNA was extracted and analyzed for the presence of viral GAG by quantitative PCR. A view of the experimental schedule is given in Fig 7B. As shown in Fig 7C, GAG was undetectable in cells treated with vehicle only. In contrast, inhibition of FOXO1 by AS1842856 led to latent proviruses recurrence in three out of four animals in a manner comparable to the positive control. The absence of reactivation in the presence of AS1842856 observed for the fourth animal was observed not only after AS1842856 treatment but also with anti-CD3/CD28, suggesting an individual response defect. To evaluate virus production obtained in these conditions, ultracentrifugated supernatants were used to infect freshly activated splenocytes from non-infected macaques. As shown in Fig 7D, five days post infection, substantial infection levels were obtained with supernatants obtained from macaques under cART treatment having shown a viral reactivation after AS1842856 treatment. These results demonstrate that inhibiting FOXO1 with AS1842856 reverses *in vivo*-induced retroviral latency leading to the production of infectious retroviral particles.

## Discussion

In this report, we show that the FOXO1 inhibitor AS1842856 induces a significant increase of both the bioenergetics and transcriptional activity of human T cells, together with a significant increase in their size, without any cell division. These modifications are accompanied by a decrease of p27 expression, contrasting with an increase of CDK2 cellular levels and by the phosphorylation of Rb and SAMHD1 proteins. As these changes are known to be characteristic of cells undergoing a G_0_ to G_1_ progression [33–35], we conclude that inhibition of FOXO1 by AS1842856 is sufficient to induce a profound reprogramming of human T lymphocytes, regulating their exit from quiescence.

Mechanisms controlling the extent of quiescence are poorly understood, representing a currently underappreciated layer of complexity in growth control [35]. When cells emerge from quiescence, they remain in the G_1_ phase of the cell cycle up to the restriction point R, defined as the point after which further progression becomes independent of continued mitogenic stimulation [34–36]. In our experiments, we observed no concomitant synthesis of DNA in T cells treated with AS1842856. This suggests that whereas FOXO1 inhibition allows T cells to progress into G_1_, it does not allow the cells to cross this restriction point to enter into S phase. These results strengthen the concept that quiescence is not a default state, but an actively maintained state [35]. They also reveal the key role played by the transcriptional program induced by FOXO1 in maintenance of quiescence in human T lymphocytes.

Upon FOXO1 inhibition, we observed an enhanced metabolic activity of T cells, affecting all T cell subsets, including naive T cells. This was illustrated by their higher expression of CD71 and CD98 metabolic markers, increased glucose uptake and greater mitochondrial respiration after AS1842856 treatment. The drug also induced a substantial cell size growth, including in naive T cells. Consistently, a comprehensive analysis of differential expression profiles of mRNA has revealed enrichment in the expression of various sets of genes involved in cell metabolism. Recent observations have shown that a hallmark of naive CD8 T cell differentiation into memory CD8 T cells is an increase of their intrinsic metabolic activity [26,37]. It is therefore tempting to speculate that inhibition of FOXO1 may not only induce G_0_ exit of naïve T cells, but also some important steps in their differentiation program into memory T cells. Thus, like quiescence, the naïve T cell state may also be actively maintained in part by FOXO1. In this context, the fact that memory T cells express lower amounts of FOXO1 is probably not fortuitous [38]. This phenomenon may also contribute to their greater responsiveness to a new antigen challenge, in keeping with the now well-established anti-proliferative action of FOXO1, in particular through specific targets of this transcription factor, such as the RhoA binding partner FAM65B [39].

One main conclusion of the present work is that reprogramming T cells after FOXO1 inhibition modifies the HIV-1/T cell interaction at several stages of the viral life cycle. We found that inhibition of FOXO1 by AS1842856 allows the efficient infection of resting T cells by HIV-1. This result is in line with our observation that the restriction factor SAMHD1 is phosphorylated after AS1842856 treatment. Indeed, it is now clear that this post-translational modification inhibits SAMHD1, the enzymatic activity of which reduces the availability of dNTP required for the viral reverse transcriptase [13]. SAMHD1 inactivation also clearly plays a role in IL-7-treated resting T cells, which are more susceptible to HIV-1 infection. IL-7 mediates signals triggering PI3-kinase activation in T cells, and an inhibition of FOXO1 in quiescent T lymphocytes after IL-7 treatment has been observed [40]. It is therefore possible that the effect of IL-7 in resting T cell infection relates to this inhibition of FOXO1, but this requires further exploration. It is quite interesting to mention here that a recent report has shown that cellular metabolism, especially glucose metabolism, seems to be a major contributor to HIV-1 reservoir implementation in CD4^+^ T cells [41]. Thus, and to explain the effect of FOXO1 inhibition on HIV-1 at the pre-integrative level, several mechanisms are likely at work. This may involve not only regulation of a group of genes required for cell cycle exit and the maintenance of cell quiescence in human T cells, like SAMHD1, but also of genes allowing a higher cell metabolism. This hypothesis is fitted very well with our transcriptomic data showing that major metabolic pathways are ranked at the top of enriched gene sets after FOXO1 inactivation by AS1842856 in PBT.

A second conclusion drawn from our results is that inhibition of FOXO1 appears to orchestrate not only pre-integrative, but also post-integrative stages of the viral life cycle. Indeed, we found an increase of viral promoter activity after AS1842856 treatment. In this case, the reactivation of LTR activity cannot be interpreted just as a consequence of some T cell activation induced by AS1842856, as this effect has been observed in TCR stimulated PBT and transformed J-Lat cells, two cellular models where cells are already very active metabolically. It has been shown that FOXO1 directly inhibits LTR activity in a TAT-dependent manner [42]. However, TAT is not expressed in the reporter system consisting of pseudotyped retrovirus encoding GFP under LTR control that we have used in PBT (Fig 5B) and in J-Lat cells (Fig 6A). Thus, we have explored the possibility that FOXO1 inhibition stimulated LTR activity indirectly. The activity of LTRs in T cells is mainly regulated by NF-κB and NFAT transcription factors. Both are inactive in resting T cells and active upon T cell stimulation. FOXO1 inhibition by AS1842856 does not affect NF-kB activity. In contrast, we found a marked activation of NFAT1. To explain this result, one explanation might be the increase basal calcium level observed in T cells after AS1842856 treatment. This finding was unexpected as no direct control of calcium homeostasis by FOXO1 has been reported to date. T-cell calcium responses to ionomycin were also strongly amplified. Interestingly, we found that Stim1 and ORAI3, two proteins controlling the entry of calcium [43], were induced at the mRNA level by AS1842856 (see S1 Table). This suggests a relationship, which still needs to be explored, between FOXO1 and the mechanisms regulating calcium fluxes in T cells. Whatever it may be, the control by FOXO1 of the NFAT pathway could be another mechanism implemented by T cells to protect them from HIV-1 infection [8]. In this context, it is interesting to note that FOXO1 is inhibited upon HIV-1 infection [15]. This is in keeping with the numerous examples of strategies that have been developed by HIV-1 to counteract the various cellular processes capable of inhibiting its viral life cycle. Therefore, FOXO1 is a central player in the interplay between HIV-1 and its cellular host.

One of the most remarkable achievements of modern biomedical research is the discovery and widespread use of cART for the treatment of HIV-1 infection. However, infected individuals who receive clinically effective antiretroviral therapy will have to continue this treatment for life. This is mainly due to the persistence of viral reservoirs, causing a plasma viral rebound observed in virtually all infected individuals who discontinue cART. The identification of compounds that can inhibit HIV-1 latency in resting CD4^+^ T cells is therefore a major challenge [5]. Our results demonstrate that AS1842856 can stimulate HIV-1 latent provirus reactivation. Therefore, AS1842856 can be considered as a LRA drug and as a new therapeutic candidate to reverse HIV-1 latency. Numerous studies reported the effects of FOXO1 inhibition by AS1842856 *in vitro* and also *in vivo* in mice [44–47]. In these studies, it appears that this drug is remarkably well tolerated, without any reported significant adverse effects, including at the immune system level. This is very encouraging with a view to its use as a therapeutic tool, alone or in combination with other pharmacological agents. Moreover, in such a shock and kill strategy, it is also possible that AS1842856 could induce some reprogramming of CD8 T-cell metabolism, thereby increasing their anti-HIV activity. The hope, behind, would be to have a treatment that will reverse latency but also capable of boosting anti-HIV immune responses, an increasingly recognized major challenge in treating this infectious disease [48].

## Methods

### Cells

The previously established J-Lat model of HIV latency was kindly provided by Eric Verdin, Gladstone Institute of Virology and Immunology. Jurkat T antigen (JTag) and J-Lat as well as HEK293T (ATCC-CRL-3216) cells were cultivated in complete RPMI medium. The H9 cell line, chronically infected with the LAI HIV-1 strain, was obtained from NIH AIDS Reagent Program. Human peripheral blood CD3 positive T lymphocytes (PBT) were purified from the blood of healthy donors as described [39]. Where indicated anti-CD3/anti-CD28-coated Dynabeads (1 beads for 5 cells, Invitrogen), IL-2 (20 U/ml, R&D Systems), or FOXO1 inhibitor AS1842856 (EMD Millipore), were used. AS1842856 was dissolved in DMSO at a 10mM stock concentration and dilutions were performed in RPMI medium. The “vehicle” condition corresponds to the same concentration of diluted DMSO or to the highest DMSO concentration used with AS1842856 in a given assay.

### Luciferase assay

JTag cells (5x10^6^) were co-transfected by electroporation (260V, 950μF) with plasmids encoding Firefly luciferase under the control of Forkhead responsive element (FRE) (1 μg), CMV-Renilla luciferase (0.1 μg), and GFP (1μg) or a constitutively active form of FOXO1 fused to GFP (FOXO1TM-GFP) (1μg). Cells were cultured in complete culture medium and 6 hours post-transfection AS1842856 or vehicle only were added. Luciferase activity was assayed 18 hours later using the Dual-Luciferase Reporter assay system (Promega) following the manufacturer’s instructions. Firefly/Renilla luciferase levels were then calculated.

### Micro-array

After validation of the RNA quality with Bioanalyzer 2100 (using Agilent RNA6000 nano chip kit), 250 ng of total RNA was reverse transcribed following the GeneChip WT Plus Reagent Kit (Affymetrix). Briefly, the resulting double strand cDNA was used for *in vitro* transcription with T7 RNA polymerase (all these steps were included in the WT cDNA synthesis and amplification kit of Affymetrix). After purification according to Affymetrix protocol, 5.5 μg of Sens Target DNA were fragmented and biotin labelled. After control of fragmentation using Bioanalyzer 2100, cDNA was then hybridized to GeneChip Clariom S Human (Affymetrix) at 45°C for 17 hours. After overnight hybridization, chips were washed on the fluidic station FS450 following specific protocols (Affymetrix) and scanned using the GCS3000 7G. The scanned images were then analyzed with Expression Console software (Affymetrix) to obtain raw data (cel files) and metrics for Quality Controls. Microarrays CEL files were directly analyzed using the Transcriptome Analysis Console software obtained from ThermoFisher Scientific. The data discussed in this publication have been deposited in NCBI's Gene Expression Omnibus and are accessible through GEO Series accession number *GSE125328* (https://www.ncbi.nlm.nih.gov/geo/query/acc.cgi?acc=GSE125328).

### Oroboros measurements of O_2_ consumption

O_2_ concentration and consumption by T cells was measured with a high-resolution respirometer (Oroboros Oxygraph-2k). Both electrodes were calibrated at 37°C and 100% oxygen before adding 2.5ml of cells (2x10^7^ cells/ml) to each chamber. After stabilization of the basal respiratory rate (*i*.*e*. in the absence of any exogenous agent) oligomycin (1μM final, Sigma Aldrich) and then successive doses of Carbonylcyanure m-chlorophénylhydrazone (CCCP, 1μM final, Sigma Aldrich) at intervals of 300 sec were added to reach the optimal concentration causing a maximal uncoupled respiratory rate.

### Western blot analysis

Protein expression levels were analyzed by Western blot as described [39]. Blotting antibodies used were anti-FOXO1 (C29H4 clone), anti-SAMHD1, anti-SAMHD1^P^ Thr592, anti-RB^P^ Ser807/811 (Cell Signaling), anti-CDK2 and anti-IκBα (Santa Cruz), anti-p27 (BD Biosciences) and anti-β-actin (Sigma), followed by HRP-conjugated goat-anti-mouse or anti-rabbit antibodies (Jackson ImmunoResearch) and ECL revelation.

### Flow cytometry

The following antibodies were used for flow cytometric analysis: anti-CD4 APC, anti-CD8 APC, anti-CD25 PE-Cy7, anti-CD127 APC and anti-Granzyme B PE (clone GB11) were from BD Biosciences. Anti-CD62-L PercP (MEL14) and anti-CD45RA FITC were from eBioscience. Biotinylated anti-CD71 and anti-CD98 were from Pharmingen and Miltenyi, respectively. Anti-GAG (clone KC57) was from Beckman Coulter. For staining with Granzyme B, GAG and SAMHD1^P^, cells were first fixed with 4% paraformaldehyde (PFA), then permeabilized in a buffer containing PBS, 1% BSA, 0.1% Triton X-100. For acridine orange staining, 10^6^ cells were washed with PBS-2% FCS at 4°C and labeled with 0.4ml of a Triton X100 0.1%, HCL 0.1 mM, NaCl 150 mM solution, followed by addition of 1.2 ml of a citric acid 0.1M, Na_2_HPO_4_ 0.2M, NaCl 150mM, EDTA 1mM solution containing 1μg/ml of acridine orange (Thermo Fischer) and directly analyzed by flow cytometry. For glucose uptake measurements, PBT treated with or without AS1842856 (500nM) for 7 days were washed twice with PBS and incubated for 45 min at 37°C with PBS, Hepes 10 mM. 2-NBDG (2-(N-(7-Nitrobenz-2-oxa-1,3-diazol-4-yl) Amino)-2-Deoxyglucose; Sigma), a fluorescent glucose analog (final concentration of 25μM), was then added and cells maintained for an additional incubation time of 30 min at 37°C. After two PBS washes, cell fluorescence was analyzed by FACS. Proliferation was assessed by dilution of CellTrace CFSE (Thermo Fisher). After two washes in PBS, cells were resuspended at 10^6^ cells/ml in a 5μM CellTrace CFSE solution and incubated at 37°C for 20 min. After loading, cells were washed with a volume of ice cold PBS 10% FCS corresponding to 5 times the loading volume. 48 hours later fluorescence was measured. Vehicle control cells stimulated with anti-CD3/CD28 coated-beads (Dynabeads, Life technologies) during 48 hours were used as a positive control of T-cell proliferation. For all experiments, fluorescence was measured on a BD FACS Calibur and analyzed using the FlowJo software.

### ImageStream flow cytometry

At the end of the culture period, cells were washed once in cold PBS and fixed for 20 minutes on ice in cytofix/cytoperm (BD Biosciences) solution. Cells were then stained with anti NFAT1 (D43B1) (Cell Signaling), and finally with anti-rabbit Alexa-488 (Cell Signaling). DAPI (Sigma D21490) (5nM) was added to stain the nucleus immediately before analyses. Flow cytometry was performed on an ImageStreamX MKII high-speed imaging flow cytometer (Amnis Corporation) and analyzed with aIDEAS Analysis Software (Amnis Corporation). To assess nuclear NFAT1 translocation, the corresponding nuclear (DAPI) image and NFAT1 (Alexa-488) image of each cell was compared and a Similarity Score (SS) was assigned for individual cells.

### Calcium Measurements

T cells were incubated for 20 min at 37°C with 1.5 μM Fura-2/AM (Molecular Probes). Experiments were performed at 37°C in mammalian saline buffer (140 mM NaCl, 5 mM KCl, 1 mM CaCl_2_, 1 mM MgCl_2_, 20 mM HEPES, 11 mM glucose). Calcium measurements by spectrofluorimetry were performed as previously described [49] with a Cary Eclipse spectrofluorimeter (Varian) (excitation: 340 and 380 nm; emission: 510 nm).

### Viral production, titration, and infection

For the production of GFP viral particles, HEK293T cells were transfected with psPAX2 lentiviral packaging plasmid along with the plasmid encoding VSV-G and HIV-1 LTR-GFP [30]. Oligonucleotides targeting firefly luciferase (5′-CGTACGCGGAATACTTCGA-3’) or FOXO1 (5-GCCGGAGTTTAGCCAGTCCAA-3’) were inserted down to H1 promoter in pSuper.Neo vector (OligoEngine) and H1-shRNA expression cassettes were introduced into the pTRIPΔU3-Gfp lentiviral vector where GFP sequence was replaced by human IL2Ralpha one. Lentiviral particles were produced and pseudotyped as previously described [50]. The titer of the virus stock was measured by flow cytometry analysis of GFP or CD25 expression, 3 days after infection of Jurkat or K562 human leukemia cells respectively. Replication-competent HIV-1 NL4.3 strains, were produced in HEK293T cells by cotransfection of the proviral plasmid in combination with pVSVg using the calcium phosphate precipitation technique as described previously [51]. The amounts of CAp24 produced were determined by enzyme-linked immunosorbent assay (ELISA; Innogenetics). 10^6^ primary cells were infected using 250 ng of CAp24 for 3 to 7 days.

### Animal infection and treatment

Four adult male cynomolgus macaques (*Macaca fascicularis* from Mauritian origin) chronically infected with SIVmac251 and treated for 60 to 75 weeks with ART were used. These macaques are part of the SIVART ANRS-IDMIT CO1 research program. Macaques were intravenously inoculated with 1,000 50% animal infectious doses (AID50) of pathogenic cell-free SIVmac251 (kindly provided by. A.M. Aubertin, Université Louis Pasteur, Strasbourg, France). 17 weeks post infection, cART regimens (kindly provided by Gilead and ViiV) were given daily at 1 ml kg^-1^ body weight by subcutaneous injections of Tenofovir disoproxyl fumarate (5.1 mg/kg), Emtricitabine (40 mg/kg) and Dolutegravir (2.5 mg/kg) [52]. Blood was periodically collected throughout the infection and the treatment for the monitoring of blood plasma viral loads, assessed as previously described [53]. Durable suppression of viremia to below the limit of quantification (37 vRNA copies/ml) was achieved after 8 weeks of treatment and was maintained during all the monitoring period. For this study, animal blood was collected after 60 (one animal), 69 (two animals) or 75 (one animal) weeks of cART treatment.

### Ethic statement

Anonymized human blood samples from the Etablissement Français du Sang (EFS, Paris, France) were obtained from healthy donors with written informed consent according to the guidelines of the medical and ethical committees of EFS and Inserm (protocol number E-2075). Experiments using human blood were performed in full compliance with French law. All experiments on non-human primates were performed under the supervision of national veterinary inspectors in accordance with French national regulations (CEA Permit Number D92-032-02) and with the Standards for Humane Care and Use of Laboratory Animals of the Office for Laboratory Animal Welfare (OLAW, USA, agreement number #A5826-01) and with European guidelines for NHP care (EU Directive N 63/2010). The study and procedures were approved by ethics committee “Comité Régional d'Ethique pour l'Expérimentation Animale Ile-De-France Sud” with notification number 15–035. Experimental procedures were performed while animals were under sedation with 10 mg/kg (body weight) of ketamine chlorhydrate and throughout the experiments all efforts were made to minimize suffering, including improved housing conditions with enrichment opportunities (12:12 light dark scheduling, provision of treats as biscuits and supplemented with fresh fruit, constant access to water supply in addition to regular play interaction with staff caregivers and research staff).

### Detection of SIV viral DNA

Cells were first purified by Ficoll-Hypaque gradient centrifugation, then CD4^+^ T cells were isolated using a CD4^+^ isolation kit (StemCell). After two days of culture with AS1842856 or anti-CD3/CD28 beads, or vehicle only, 3x10^6^ CD4^+^ T cells were co-cultured with 10^6^ activated heterologous simian splenocytes for nine days. SIV DNA quantifications were performed as in Ponte et al. [54]. Cells were lysed in Tween-20 (0.05%), Nonidet P-40 (0.05%), and proteinase K (100μg/ml) for 30min at 56°C, followed by 15min at 98°C. *Gag* sequences were amplified together with the rhesus macaque CD3γ chain in triplicate using the “outer” 3′/5′ primer pairs by 15min of denaturation at 95°C, followed by 22 cycles of 30sec at 95°C, 30sec at 60°C, and 3min at 72°C. *SIV-Gag* and CD3γ were quantified within each of the PCR products in LightCycler experiments performed on 1/280th of the PCR products; “inner” 3′/5′ primer pairs and the LightCycler480 SYBR Green I Master Mix (Roche Diagnostics, Meylan, France) were used. The PCR cycling program consisted of 10min of initial denaturation at 95°C, 40 cycles of 10sec at 95°C, 6sec at 64°C, and 15sec at 72°C. Fluorescence measurements were performed at the end of the elongation steps. Plasmids containing one copy of both the CD3γ and *SIV*-*Gag* amplicons were used to generate standard curves. Quantifications were performed in independent experiments using the same first-round serial dilution standard curve. Quantifications were made in triplicate for all samples studied. The sequences of primers used were CD3-Out-5’: ACTGACATGGAACAGGGGAA, CD3-Out-3’: AGCTCTGAAGTAGGGAACATAT, *SIV-Gag*-Out-5’: CAACAAGGACAGCTTAGGGA, *SIV-Gag* -Out-3’: TTGACAGGCCGTCAGCATTT, CD3-In-5’: GGCTATCATTCTTCTTCAAGGTA, CD3-In-3’: TTCCTGGCCTATGCCCTTTT, *SIV-Gag*-In-5’: CCGTCAGGATCAGATATTGCA, *SIV-Gag* -In-3’: GAAACTATGCCAAAAACAAGT. The results were expressed as the absolute number of SIV copies per 10^5^ cells.

In these experiments, at the end of the culture, supernatants were collected and passed through 0.45-μm pore filters. Viral particles were then concentrated through a 25% sucrose cushion by ultracentrifuged at 150000 x g for 1 h. Concentrated viruses were then added to 10^6^ heterologous splenocytes from non-infected monkey preactivated with anti-CD3/CD28 beads. After five days of culture, cells were harvested and infection levels were measured by *Gag* sequence quantification in genomic DNA as described above.

### Statistical analysis

Means +/- SE are shown when indicated. Statistically significant differences between groups were assessed with the Graph Prism software using Student’s *t* tests. (*p < 0.05; **p < 0.01; ***p < 0.001).

## Supporting information

S1 Table Transcriptome analysis of PBT exposed to AS1842856 treatment. PBT from 3 individual donors were cultured during 7 days in the presence of AS1842856 (500 nM) or vehicle only. Total RNA from these cells was reverse transcribed, resulting cDNAs were biotin labelled, then hybridized to Affymetrix GeneChip Clariom S Human arrays. Scanned images of these arrays were then analyzed with Expression Console software (Affymetrix) to obtain raw data (cel files) and metrics for Quality Controls. Microarrays CEL files were directly analyzed using the Transcriptome Analysis Console software (from ThermoFisher Scientific). Two distinct gene lists were established on the basis of a <-1.5 and >1.5-fold change cut-off and a P-val <0.01 between samples treated with A1842856 or vehicle only.
(XLSX)

S1 Fig AS1842856 allows HIV-1 infection of human resting T cells in a dose dependent manner. PBT were cultured with increased concentrations of AS1842856. After 7 days, cells were infected with the HIV-1 strain NL4.3 pseudotype (upper panel) or with LAI virus (lower panel). After 3 days of infection, GAG expression was measured by FACS using a GAG-specific antibody. Mean results +/- SE with cells from 3 different donors are shown.
(PDF)

S2 Fig AS1842856 induces significant T-cell size increase in all T cell subsets. (A) FSC of PBT treated with AS1842856 (500nM) or vehicle only were analyzed by FACS at different time points during 7 days of culture. Mean results +/- SE from 5 independent donors are shown. (B) PBT were cultured for 7 days with various concentrations of AS1842856 or the corresponding dilution of vehicle. (C) After 7 days of treatment with AS1842856 (500nM) or vehicle only, a total cell count of the viable cells in the culture was performed (mean results +/- SE with cells from five different donors). (D) PBT were cultured for 7 days with 500nM of AS1842856 or vehicle only; FSC of CD45RA-positive (naïve) and CD45RA-negative (memory) sub-populations was then measured by FACS after labeling with CD4, CD8 and CD45RA-specific antibodies. Mean results +/- SE from 6 independent donors are shown.
(PDF)

S3 Fig AS1842856 does not initiate proliferation of PBT. PBT were cultured for 7 days with AS1842856 (500 nM) or vehicle only, then stained with CFSE and stimulated or not for 48 hrs with anti-CD3/CD28 coated beads. Cell fluorescence was analyzed by FACS. Result obtained with one representative donor (upper panel) and mean results +/- SE with T cells from 3 independent donors (lower panel) are shown.
(PDF)

S4 Fig Both AS1842856 and TCR stimulation lead to SAMHD1 phosphorylation. PBT were cultured for 7 days with AS1842856 (500nM) or vehicle only. A parallel stimulation with anti-CD3/CD28 coated beads was also performed as indicated. Cells were then collected, lysed and immunoblotted using specific antibodies directed to the phosphorylated form of SAMHD1 and β-actin as a control (upper panel). Blot quantification of SAMHD1 phosphorylation, +/- SE, with cells from two different donors are shown in the lower panel. Data were normalized for values obtained with β-actin blots.
(PDF)

S5 Fig Infection of AS1842856-treated PBT correlates with SAMHD1 phosphorylation levels. PBT from heathy donors were cultured with AS1842856 (500nM) or vehicle only for 7 days and infected with the HIV-1 strain NL4.3. After 3 days of infection, SAMHD1 phosphorylation was measured by FACS in the GAG positive (infected) and GAG negative (non-infected)-gated cells populations. Results obtained with one representative donor are shown in the left panel and mean results, +/- SE, with cells from three different donors in the right panel.
(PDF)

S6 Fig IκBα protein levels are not affected by AS1842856. PBT were cultured for 7 days with AS1842856 (500nM) or vehicle only and then stimulated or not with PMA plus ionomycin as indicated. After 30 min of stimulation, cells were collected, lysed and immunoblotted using specific antibodies against IκBα and β-actin as a control (upper panel). Results of blot quantification, +/- SE, with cells from two different donors are shown in the lower panel. Data were normalized for values obtained with β-actin blots.
(PDF)

S7 Fig AS1842856 potentiates calcium responses. PBT were cultured in the presence of AS1842856 (500nM) or vehicle only for 7 days. Levels of intracellular calcium were measured by spectrofluorometry using the calcium fluorescent indicator Fura-2 at the steady state (A) or after ionomycin (500nM) stimulation (B). Mean results +/- SE of calcium responses obtained from 6 and 3 independent donors are shown in A and B, respectively.
(PDF)

S8 Fig AS1842856 inhibits FOXO1 transcriptional activity in the Jurkat T cell model. (A) The promoter activity of the Forkhead responsive element (FRE) was measured using a dual luciferase assay in Jurkat JTag cells transfected with vectors encoding either GFP or a constitutively active form of FOXO1 (FOXO1TM GFP) together with luciferase reporter plasmids (FRE-Firefly luciferase and CMV-Renilla luciferase), and then treated for 18 hrs with various concentrations of AS1842856 or vehicle only. Mean results +/- SE from 3 independent experiments are shown. (B) JTag cells were transfected with vectors encoding either GFP or a constitutively active form of FOXO1 (FOXO1TM GFP) and cultured in the presence of various concentrations of AS1842856 or vehicle only. 3 days later CD62-L cell surface expression was measured by FACS on the GFP-positive gated cell populations. Mean results +/- SE from 4 independent experiments are shown.
(PDF)

S9 Fig AS1842856 allows HIV-1 latent provirus reactivation in the J-Lat HIV-1 latency model. J-Lat cell clone A2 and A7 were incubated with increased concentrations of AS1842856. After three days of culture, the percentage of GFP-positive cells was measured by FACS. Mean results +/- SE from 3 independent experiments are shown.
(PDF)
