# Supplementary figures and images for: FOXO1 transcription factor plays a key role in T cell—HIV-1 interaction
Source: PLoS Pathog. 2019 May 1;15(5):e1007669. doi: 10.1371/journal.ppat.1007669 (PMC6513100; doi:10.1371/journal.ppat.1007669)

# S1 Figure

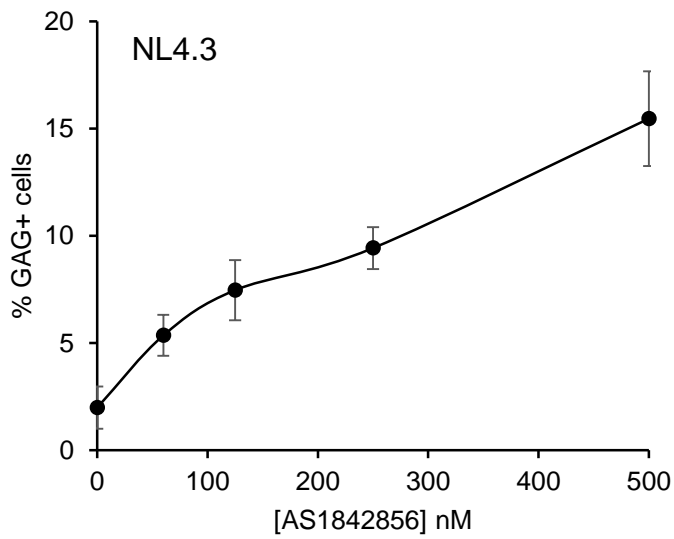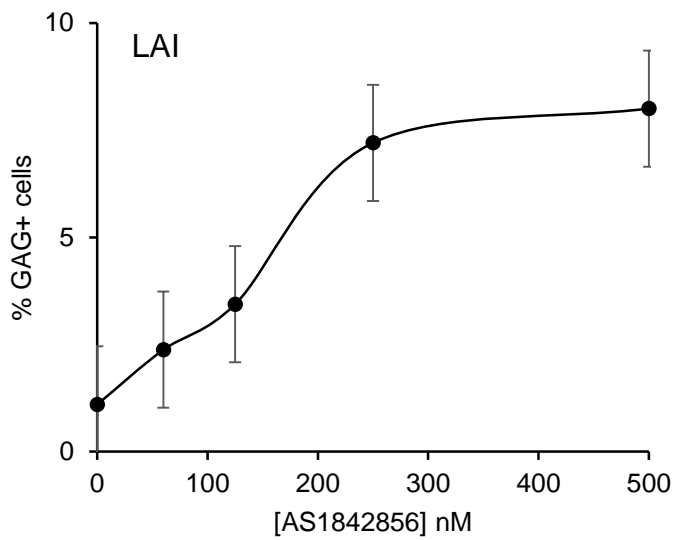

Supplement: S1 Fig — PBT were cultured with increased concentrations of AS1842856. After 7 days, cells were infected with the HIV-1 strain NL4.3 pseudotype (upper panel) or with LAI virus (lower panel). After 3 days of infection, GAG expression was measured by FACS using a GAG-specific antibody. Mean results +/- SE with cells from 3 different donors are shown. (PDF) [file ppat.1007669.s002.pdf]

## S2 Figure

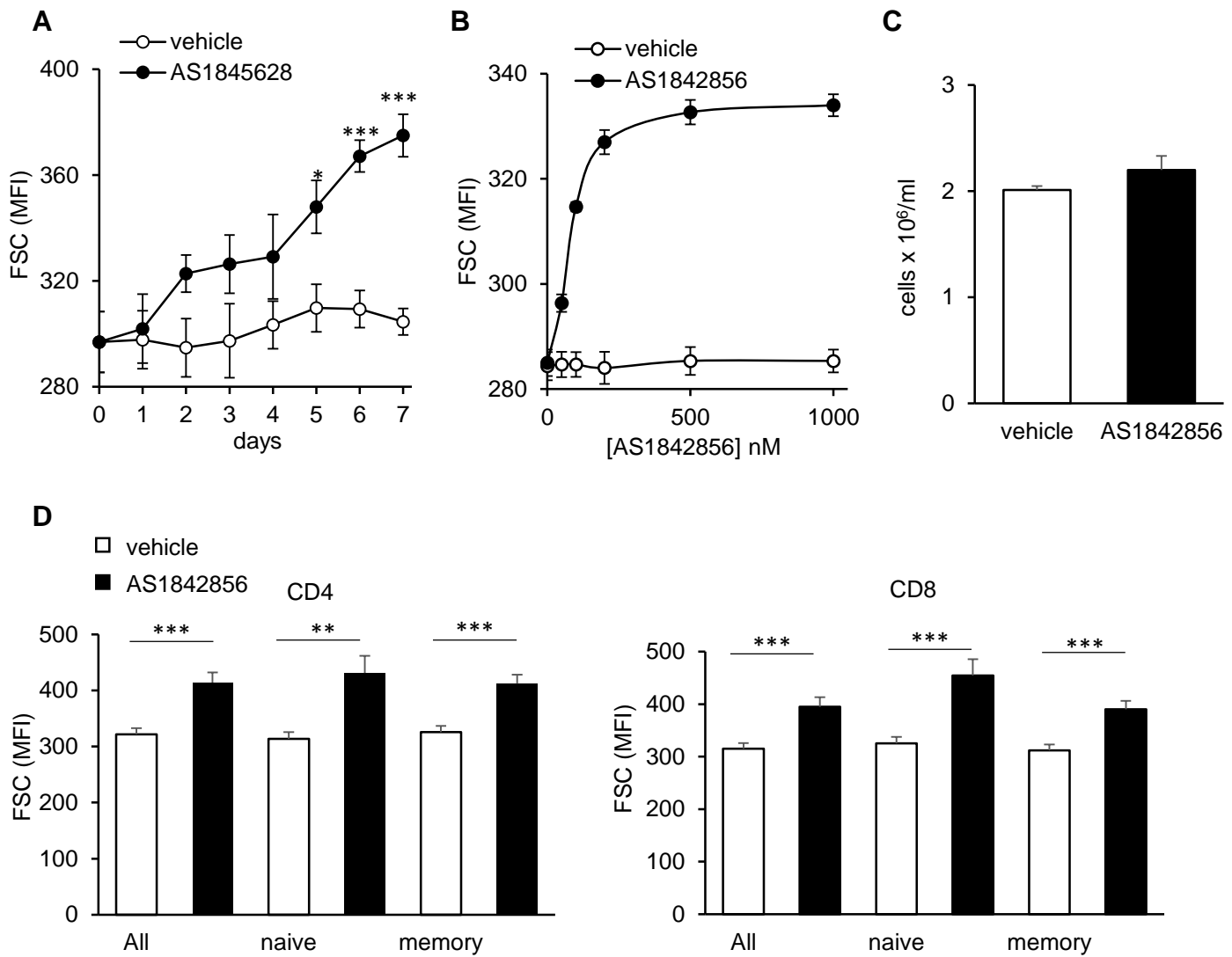

Supplement: S2 Fig — (A) FSC of PBT treated with AS1842856 (500nM) or vehicle only were analyzed by FACS at different time points during 7 days of culture. Mean results +/- SE from 5 independent donors are shown. (B) PBT were cultured for 7 days with various concentrations of AS1842856 or the corresponding dilution of vehicle. (C) After 7 days of treatment with AS1842856 (500nM) or vehicle only, a total cell count of the viable cells in the culture was performed (mean results +/- SE with cells from five different donors). (D) PBT were cultured for 7 days with 500nM of AS1842856 or vehicle only; FSC of CD45RA-positive (naïve) and CD45RA-negative (memory) sub-populations was then measured by FACS after labeling with CD4, CD8 and CD45RA-specific antibodies. Mean results +/- SE from 6 independent donors are shown. (PDF) [file ppat.1007669.s003.pdf]

S3 Figure

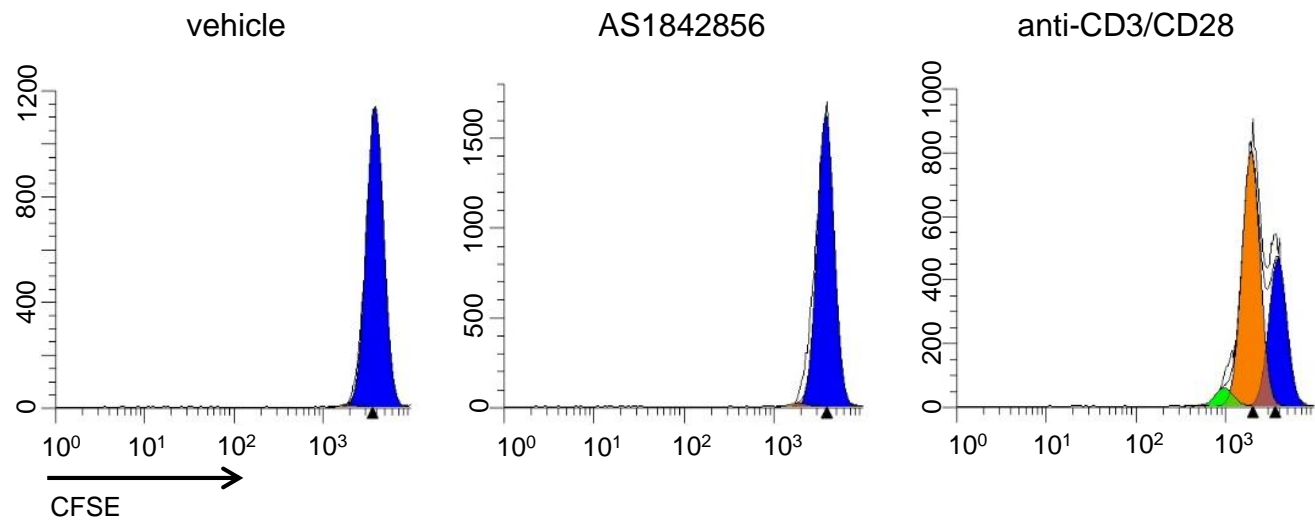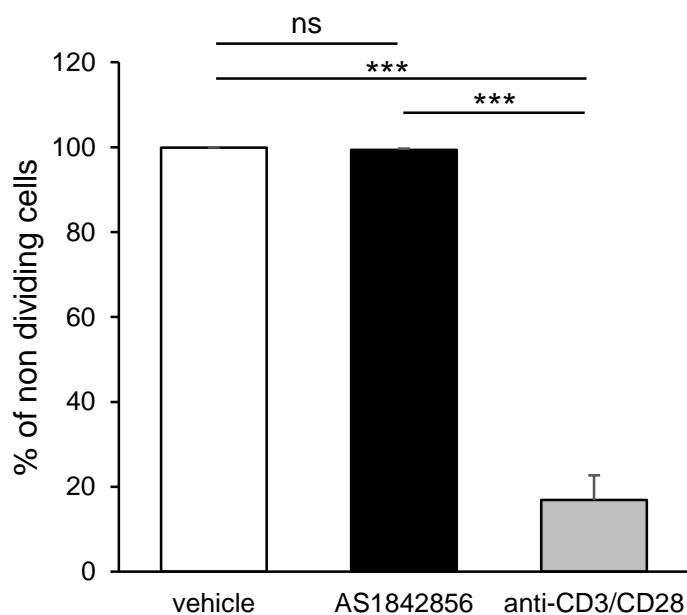

Supplement: S3 Fig — PBT were cultured for 7 days with AS1842856 (500 nM) or vehicle only, then stained with CFSE and stimulated or not for 48 hrs with anti-CD3/CD28 coated beads. Cell fluorescence was analyzed by FACS. Result obtained with one representative donor (upper panel) and mean results +/- SE with T cells from 3 independent donors (lower panel) are shown. (PDF) [file ppat.1007669.s004.pdf]

# S4 Figure

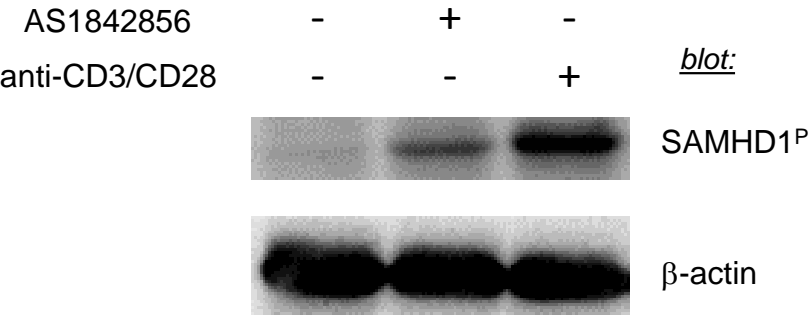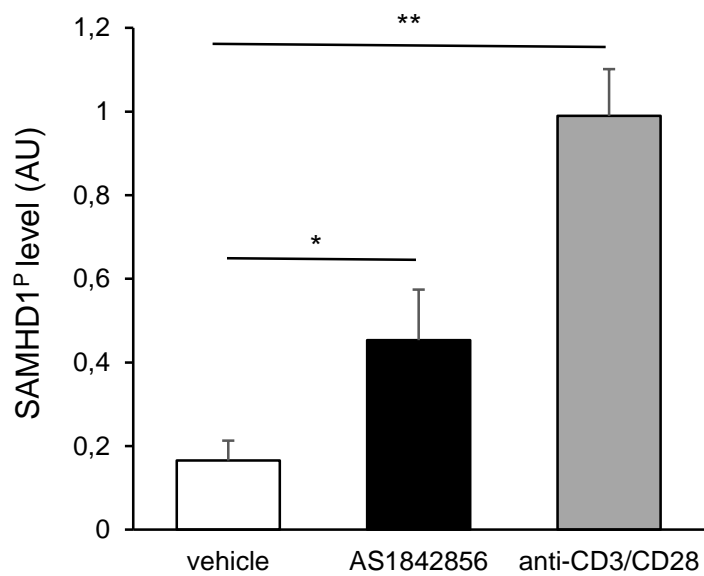

Supplement: S4 Fig — PBT were cultured for 7 days with AS1842856 (500nM) or vehicle only. A parallel stimulation with anti-CD3/CD28 coated beads was also performed as indicated. Cells were then collected, lysed and immunoblotted using specific antibodies directed to the phosphorylated form of SAMHD1 and β-actin as a control (upper panel). Blot quantification of SAMHD1 phosphorylation, +/- SE, with cells from two different donors are shown in the lower panel. Data were normalized for values obtained with β-actin blots. (PDF) [file ppat.1007669.s005.pdf]

S5 Figure

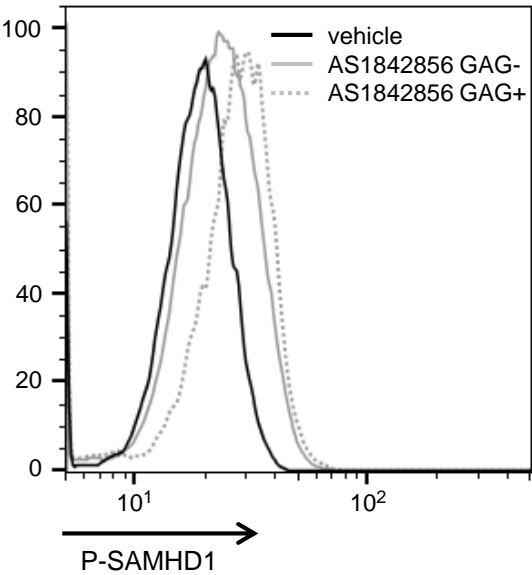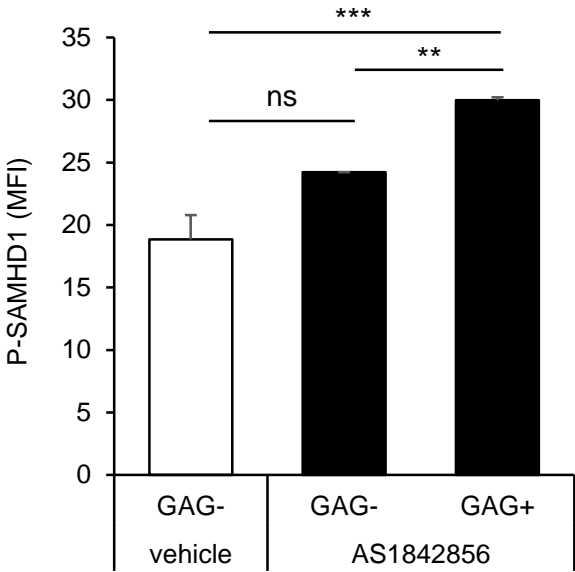

Supplement: S5 Fig — PBT from heathy donors were cultured with AS1842856 (500nM) or vehicle only for 7 days and infected with the HIV-1 strain NL4.3. After 3 days of infection, SAMHD1 phosphorylation was measured by FACS in the GAG positive (infected) and GAG negative (non-infected)-gated cells populations. Results obtained with one representative donor are shown in the left panel and mean results, +/- SE, with cells from three different donors in the right panel. (PDF) [file ppat.1007669.s006.pdf]

S6 Figure

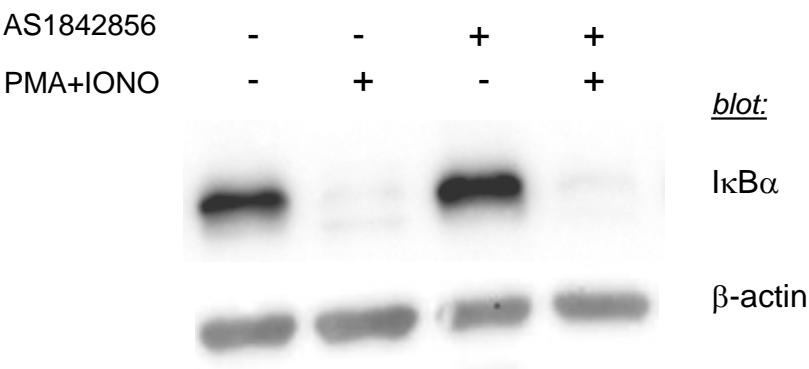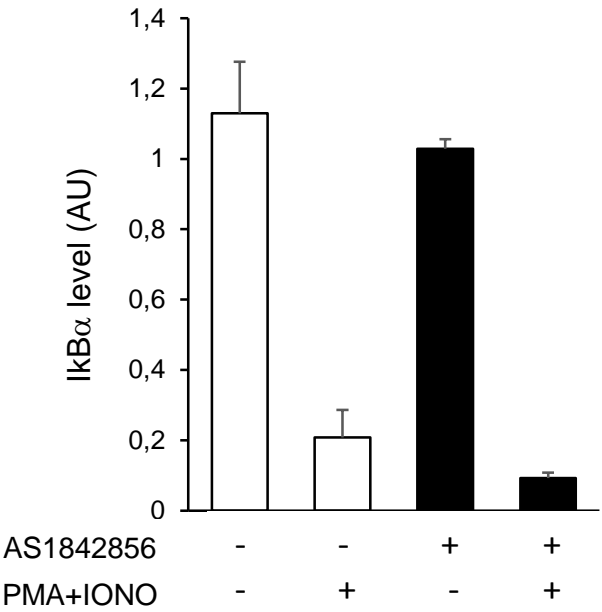

Supplement: S6 Fig — PBT were cultured for 7 days with AS1842856 (500nM) or vehicle only and then stimulated or not with PMA plus ionomycin as indicated. After 30 min of stimulation, cells were collected, lysed and immunoblotted using specific antibodies against IκBα and β-actin as a control (upper panel). Results of blot quantification, +/- SE, with cells from two different donors are shown in the lower panel. Data were normalized for values obtained with β-actin blots. (PDF) [file ppat.1007669.s007.pdf]

# S7 Figure

A

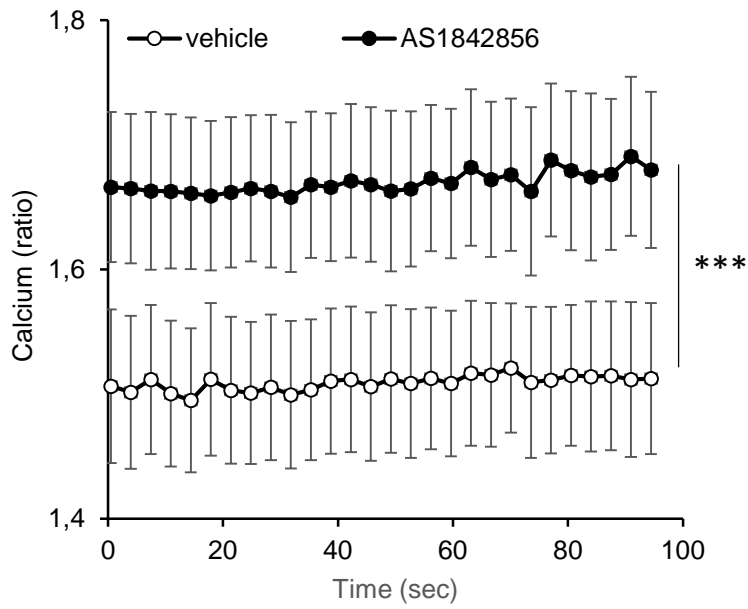

B

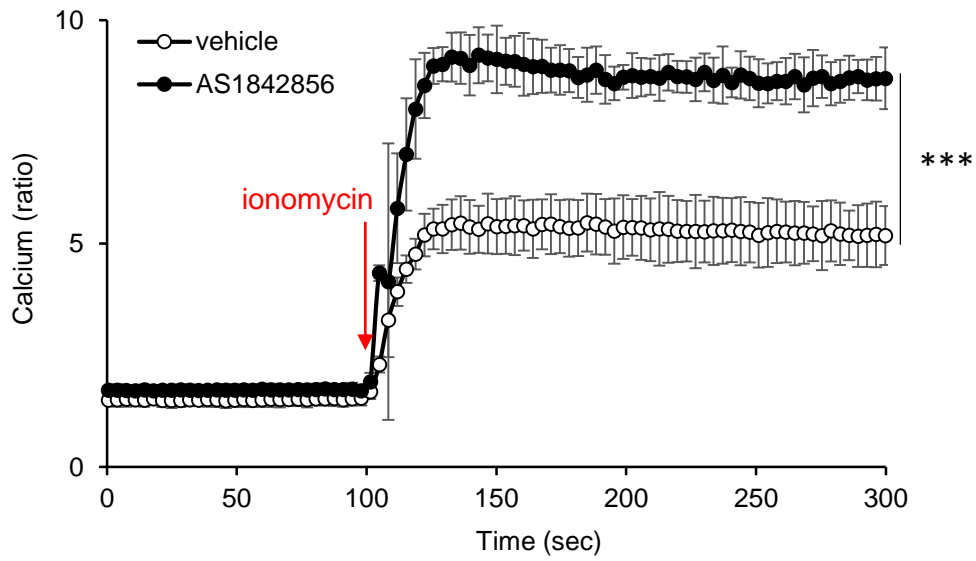

Supplement: S7 Fig — PBT were cultured in the presence of AS1842856 (500nM) or vehicle only for 7 days. Levels of intracellular calcium were measured by spectrofluorometry using the calcium fluorescent indicator Fura-2 at the steady state (A) or after ionomycin (500nM) stimulation (B). Mean results +/- SE of calcium responses obtained from 6 and 3 independent donors are shown in A and B, respectively. (PDF) [file ppat.1007669.s008.pdf]

S8 Figure

A

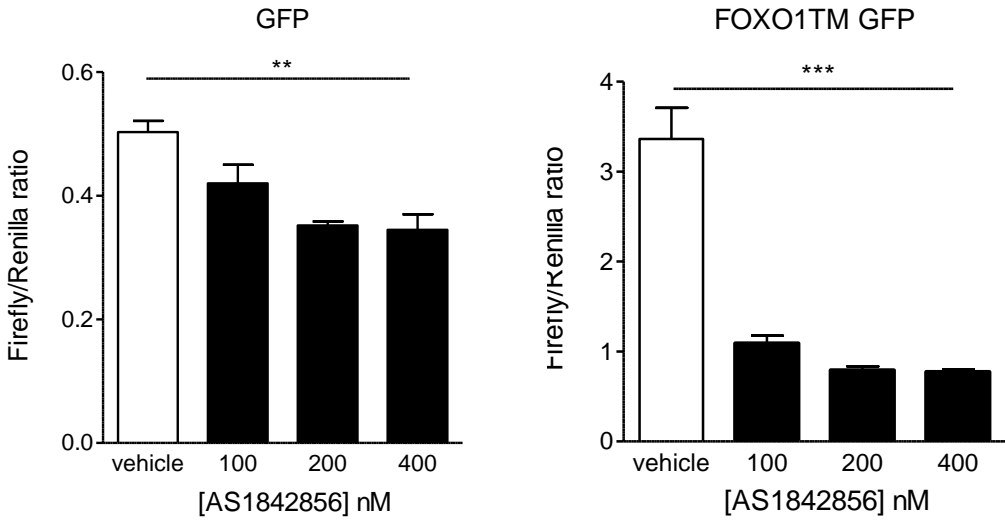

B

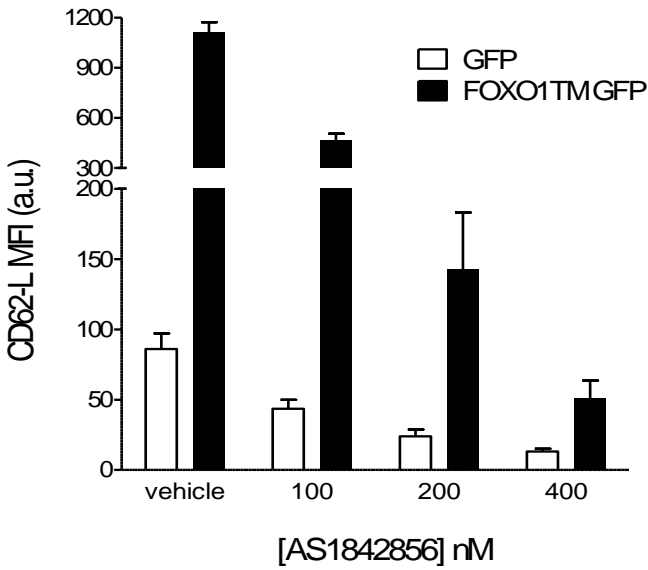

Supplement: S8 Fig — (A) The promoter activity of the Forkhead responsive element (FRE) was measured using a dual luciferase assay in Jurkat JTag cells transfected with vectors encoding either GFP or a constitutively active form of FOXO1 (FOXO1TM GFP) together with luciferase reporter plasmids (FRE-Firefly luciferase and CMV-Renilla luciferase), and then treated for 18 hrs with various concentrations of AS1842856 or vehicle only. Mean results +/- SE from 3 independent experiments are shown. (B) JTag cells were transfected with vectors encoding either GFP or a constitutively active form of FOXO1 (FOXO1TM GFP) and cultured in the presence of various concentrations of AS1842856 or vehicle only. 3 days later CD62-L cell surface expression was measured by FACS on the GFP-positive gated cell populations. Mean results +/- SE from 4 independent experiments are shown. (PDF) [file ppat.1007669.s009.pdf]

S9 Figure

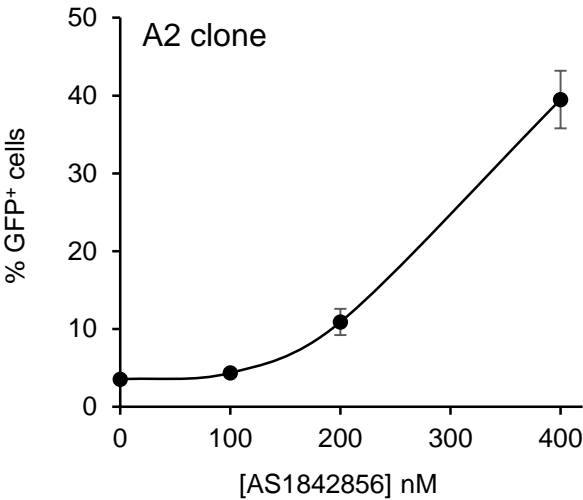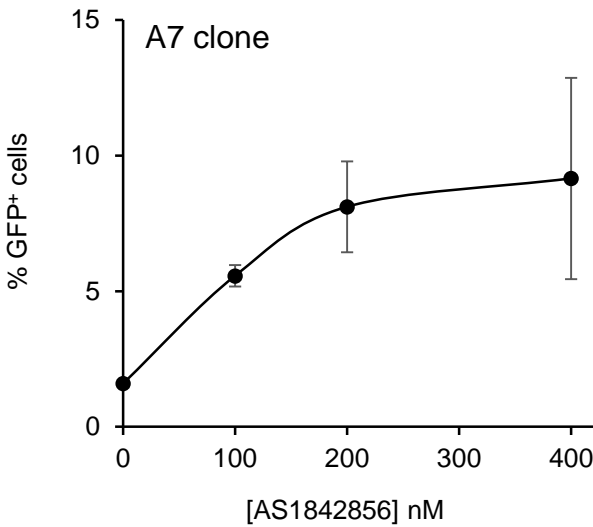

Supplement: S9 Fig — J-Lat cell clone A2 and A7 were incubated with increased concentrations of AS1842856. After three days of culture, the percentage of GFP-positive cells was measured by FACS. Mean results +/- SE from 3 independent experiments are shown. (PDF) [file ppat.1007669.s010.pdf]
